# Heterobimetallic Cu–*Ln* complexes with sulfonyl­amido­phosphate and Schiff base ligands: synthesis and structure

**DOI:** 10.1107/S2056989026003002

**Published:** 2026-04-14

**Authors:** Mariia B. Struhatska, Viktor O. Trush, Olesia V. Moroz, Irina S. Konovalova, Svitlana V. Shishkina, Volodymyr M. Amirkhanov

**Affiliations:** a Department of Chemistry, Taras Shevchenko National University of Kyiv, 12, Hetman Pavlo Skoropadsky Str., Kyiv 01033, Ukraine; b Institut für Anorganische Chemie und Strukturchemie Heinrich-Heine-University, Düsseldorf, Universitätsstr 1 40224 Düsseldorf Germany; c Institute of Organic Chemistry of the National Academy of Sciences of Ukraine, Akademika Kukharya Str. 5, Kyiv 02660, Ukraine; Universidade Federal do ABC, Brazil

**Keywords:** crystal structure, sulfonyl­amido­phosphate, 3*d*–4*f* complex, Schiff base

## Abstract

The synthesis and structural characterization of two new heterobimetallic 3*d*–4*f* complexes is reported, namely, bis­[dimethyl (phenyl­sulfon­yl)amido­phosphato]-μ-nitrato-(μ-6,6′-{(1*E*,1′*E*)-[propane-1,3-diylbis(aza­nylyl­idene)]bis­(methanylyl­idene)}bis­(2-meth­oxy­phenolato))cerium(III)copper(II), [CeCu(C_19_H_20_N_2_O_4_)(C_8_H_11_NO_5_PS)_2_(NO_3_)] or [Cu(Vanpen)Ce(μ-NO_3_)(*L*)_2_] (**1**), and [μ-dimethyl (phenyl­sulfon­yl)amido­phosphato][dimeth­yl(phenyl­sulfon­yl)amido­phosphato](nitrato)(μ-6,6′-{(1*E*,1′*E*)-[propane-1,3-diylbis(aza­nylyl­idene)]bis­(methanylyl­idene)}bis­(2-meth­oxy­phenolato))gadolinium(III)copper(II), [CuGd(C_19_H_20_N_2_O_4_)(C_8_H_11_NO_5_PS)_2_(NO_3_)] or [Cu(Vanpen)Gd(μ-*L*)(*L*)(NO_3_)] (**2**), obtained by combining the Schiff base (6,6′-{(1*E*,1′*E*)-[propane-1,3-diylbis(aza­nylyl­idene)]bis­(methanylyl­idene)}bis­(2-meth­oxy­phenol)) complex Cu(Vanpen) with the lanthanide(III) nitrates and sulfonyl­amido­phosphate (SAPh) ligand dimeth­yl(phenyl­sulfon­yl)amido­phosphate (*L*^−^). Single-crystal X-ray diffraction studies of Cu–Ce and Cu^2+^–Gd^3+^ complexes reveal that these species contain the *Ln*(*L*)_2_NO_3_ unit. The lanthanide centers adopt nine-coordinate, distorted muffin (*Cs*) geometries. The coordination modes of *L*^−^ and NO^3−^ ligands change depending on the lanthanide. In the Cu^2+^–Ce^3+^ complex the NO_3_^−^ group acts as a bridging ligand, whereas in the Cu^2+^–Gd^3+^ analogue the bridging function is assumed by SAPh, with NO_3_^−^ binding only in a chelating fashion. The presence of a nitrate group can be explained by steric hindrance that prevents coordination of three SAPh ligands around the lanthanide center. Qualitative X-ray fluorescence analysis confirmed the presence of two different metal ions within each heterometallic species.

## Abstract 2

A strategy of combining a Cu^2+^–Schiff base unit with a sulfonyl­amido­phosphate co-ligand and lanthanide(III) nitrate enabled the formation of two new heterobimetallic 3*d*–4*f* complexes. Structural analysis of the Cu^2+^–Ce^3+^ and Cu^2+^–Gd^3+^ compounds reveals distinct coordination modes of the sulfonyl­amido­phosphate and NO_3_^−^ ligands and a geometry-dependent rearrangement of the bridging fragments. These findings suggest that steric effects, together with subtle differences in metal–ligand inter­actions, govern the assembly of Cu^2+^–*Ln*^3+^ units and influence structural parameters relevant to 3*d*–4*f* exchange pathways.

## 1. Chemical context

The application of a ligand strategy based on Schiff bases leads to inter­mediate complexes with any first-row transition metal. Some of these metals, together with 4*f* ions, can contribute to anisotropy in new complexes (Chandrasekhar *et al.*, 2007 ▸; Kotrle *et al.*, 2021 ▸). This, combined with the inherently high spin of the mol­ecule, is a key prerequisite for the appearance of single-mol­ecule magnetic behavior (Sutter *et al.*, 2008 ▸). An important ongoing task is the synthesis of complexes with varied geometries to assess how these structural changes influence the magnitude of the 3*d*–4*f* exchange-inter­action parameter. The presence of additional meth­oxy donor groups in salen-type Schiff base ligands such as H_2_Vanpen provides suitable coordination sites for binding a lanthanide ion to the pre-formed 3*d* complex, thereby enabling the assembly of the heterometallic 3*d*–4*f* system.
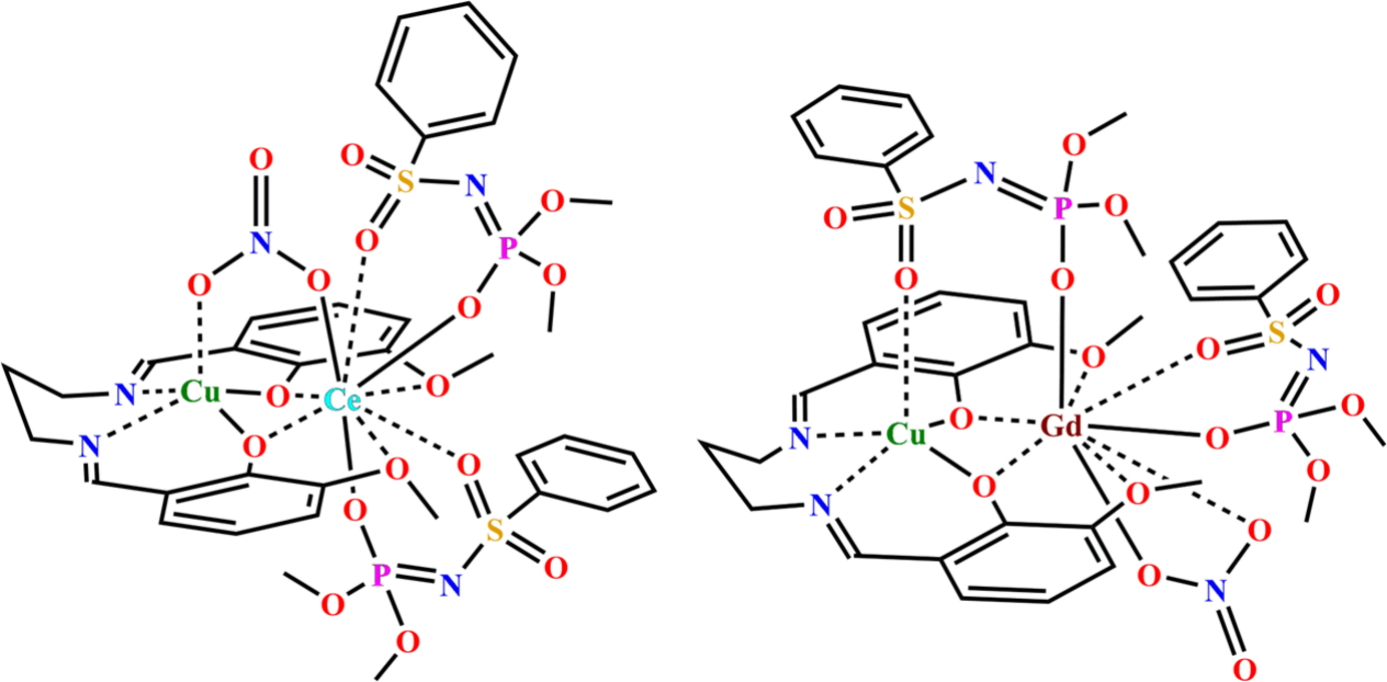


The magnitude of the magnetic exchange inter­action parameter (*J*) correlates with the value of the torsion angle (δ) defined between the *M*O_*n*_O_*n*+1_ and *Ln*O_*n*_O_*n*+1_ planes within the bridging *M*O_2_*Ln* fragment. A decrease in the δ angle promotes stronger ferromagnetic coupling between the 3*d* and 4*f* metal centers. Based on the reported δ and *J* values for previously studied Cu–Gd systems (Costes *et al.*, 1996 ▸, 1998 ▸, 2000 ▸; Ryaza­nov *et al.*, 2002 ▸), complex **2** described in this work can be regarded as a promising candidate for magnetochemical investigations. This assumption is supported by the exceptionally small dihedral angle observed in its structure [δ = 4.00 (9)°], indicating a nearly planar CuO_2_Gd bridging core that is favorable for efficient magnetic exchange.

## 2. Structural commentary

**Figure 1:**
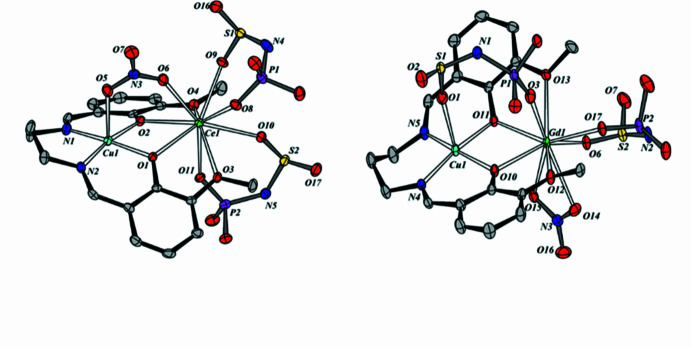
Mol­ecular structures of **1** (left panel) and **2** (right panel) (H atoms, meth­oxy group carbon atoms, and phenyl rings are omitted in the illustration).

The crystal structures of both complexes consist of heterobimetallic mol­ecules containing a neutral Cu(Vanpen) fragment and a coordinated *LnL*_2_NO_3_ moiety. The Ce^3+^–Cu^2+^ and Gd^3+^–Cu^2+^ metal centres are linked by two bridging phenolate oxygen atoms from (Vanpen)^2−^ and by either a nitrate group (**1**) or a deprotonated SAPh ligand (**2**) (Fig. 1 ▸). The Cu⋯Ce and Cu⋯Gd separations are 3.5334 (6) and 3.4939 (5) Å, respectively. The N_2_O_2_ chelating plane of the (Vanpen)^2−^ ligand coordinated to Cu^2+^ is formed by two phenolate oxygen atoms and two imine nitro­gen atoms. The Cu^2+^ centres adopt tetra­gonal-pyramidal O_3_N_2_ coordination environments, as indicated by the calculated trigonality indices τ = 0.012 and τ = 0.112 for complexes **1** and **2**, respectively [τ = (β - α)/60, with α = O3—Cu1—N1 = 169.67 (15)°, β = O2—Cu1—N2 = 170.36 (14)°; α = O2—Cu1—N1 = 165.91 (15)°, β = O3—Cu1—N2 = 172.60 (13)°; see Tables 1 ▸ and 2 ▸) (Addison *et al.*, 1984 ▸). In complex **1** the coordination polyhedron is essentially ideal, whereas in **2** it is slightly distorted. The apical position of the tetra­gonal pyramid is occupied by an oxygen atom of the nitrate group (**1**) or of the sulfonyl moiety of the phospho­ramidate ligand *L*^−^ (**2**), which also acts as a bridge between the Ce^3+^–Cu^2+^ and Gd^3±^–Cu^2+^ centres, respectively. The average Cu1—N bond lengths are 1.974 and 1.976 Å for complexes **1** and **2**. The Cu1—O15 (**1**) and Cu1—O5 (**2**) distances to the bridging nitrate and *L*^−^ ligands occupying the apical position are 2.329 (3) and 2.372 (3) Å, respectively (Tables 1 ▸ and 2 ▸).

The planarity of the Cu(Vanpen) fragment in both complexes is described by inter­planar and dihedral angles summarized in Tables 3 ▸ and 4 ▸. The CuO_2_Gd fragment is almost planar, as indicated by the torsion angle Cu1—O2—O3—Gd1 = 4.00 (9)deg;. The maximum deviation of the atoms from the corresponding least-squares plane is 0.059 Å. In contrast to this nearly planar arrangement, the CuO_2_Ce four-membered fragment is more distorted: the maximum atomic deviation from the Cu1–O2–O3–Ce1 plane is 0.252 (3) Å, and the torsion angle is 17.49 (8)°. The maximum deviation of the atoms from the least-squares plane defined by the CuO_2_N_2_ fragment is 0.179 (4) Å for complex **1** and 0.227 (4) Å for complex **2**. The C9 atoms in **1** and **2** lie above the plane formed by the imine nitro­gen atoms and the copper atom by 1.298 (5) and 0.42 (5) Å, respectively. The Cu(Vanpen) fragment in heterobimetallic complexes **1** and **2** adopts a butterfly conformation along the O2⋯O3 hinge lines, respectively. The angles between the planes Cu1/N1/O2 and Cu1/N2/O3 are 10.88 (14)° for **1** and 11.44 (12) ° for **2** (Tables 3 ▸ and 4 ▸). The copper atoms are displaced from the C7/N1/N2/C11 planes in **1** and **2** by 1.2225 (5) and 0.8045 (5) Å, respectively. The coordination number of the Ce^3+^ and Gd^3+^ ions is 9. The coordination polyhedra of Ce in complex **1** and Gd in complex **2** were evaluated using the *Shape 2.1* program (Llunell *et al.*, 2013 ▸) and identified as distorted muffin (*Cs*) geometries formed by nine oxygen atoms: four derived from the Cu(Vanpen) fragment and five from the deprotonated sulfamidate ligands and the nitrate group. The deviation from ideal symmetry is greater for the Gd-containing complex. The coordination behaviour of these ligands differs for Ce^3+^ and Gd^3+^. In the Ce^3+^ complex, two SAPh ligands coordinate in the classical (*O*,*O*′)-chelating mode, while the ninth position is occupied by an oxygen atom of the bridging NO_3_^−^ group. In the Gd^3+^ complex, one SAPh ligand coordinates in a bidentate-cyclic fashion through the phosphoryl and sulfonyl oxygen atoms, the NO_3_^−^ anion chelates only the Gd^3+^ center, and the ninth position is occupied by the phosphoryl oxygen atom of the second SAPh ligand, which acts as a bridge. The Ce1—O and Gd1—O bond lengths fall within the ranges 2.437 (3)–2.716 (3) and 2.284 (3)–2.578 (3)Å, respectively. The longest distances correspond to meth­oxy oxygen atoms: Ce1—O1 = 2.716 (3) Å and Ce1—O4 = 2.661 (3) Å; for Gd1, the corresponding values are Gd1—O1 = 2.570 (3) Å and Gd1—O4 = 2.578 (3) Å (Tables 2 ▸ and 3 ▸). The average Ce—O(P) and Gd—O(P) as well as Ce—O(S) and Gd—O(S) bond lengths to the phosphoryl and sulfonyl groups of the cyclically chelating SAPh ligand are 2.477 and 2.400 Å, respectively. For the bridging SAPh ligand in complex **2**, the Gd—O(P) distance is 2.284 (3) Å. The Gd—O15 and Gd—O16 bond lengths are 2.475 (3) and 2.508 (3) Å, comparable to those observed in heterometallic lanthanide nitrate complexes where NO_3_^−^ acts as a bidentate-cyclic ligand: *d*_avg_[Gd—O(N)] = 2.59 and 2.58 Å in [Zn(Vanen)Ce(NO_3_)_3_] and [Zn(Vanen)Ce(NO_3_)_3_·CH_3_OH], respectively (Sui *et al.*, 2007 ▸). In complex **1**, the Ce—O(N) distance to the μ-NO_3_^−^ group is 2.573 (3) Å (compared to 2.457 Å in the Zn–Ce analogue).

In complex **1**, the two *L*^−^ ligands form six-membered chelate metallacycles upon coordination to the Ce^3+^ center. One of these rings is nearly planar [torsion angle Ce1—O5—O7—N3 = 2.74 (14)°], whereas the other is more distorted [∠Ce1—O10—O12—N4 = 8.35 (14)°]. The conformation of the O–S–N–P–O fragment is distorted due to the deviation of the coordinated sulfonyl group relative to the N4—P2 bond [torsion angle O10—S2—N4—P2 = −39.9 (4)°]. For the atom sets O5/S1/N3/P1/O7 and O10/S2/N4/P2/O12, the maximum deviations from the least-squares planes do not exceed 0.125 (3) and 0.295 (5) Å, respectively. The bidentate-bridging nitrate group is essentially planar, with a maximum deviation from the least-squares plane through O15, N5, O16, O17 of only 0.005 (4) Å. Deprotonation of H*L* leads to an increase in the P—O and S—O bond lengths of 0.027 and 0.047 Å [*d*(P—O)_lig_ = 1.456 Å; *d*_avg_(S—O)_lig_ = 1.423Å] and to a shortening of the S—N and P—N bonds by 0.102 and 0.075 Å [*d*(S—N)_lig_ = 1.642 Å; *d*(P—N)_lig_ = 1.662 Å] within the O–S–N–P–O node. In the coordinated nitrate, the N5—O17 bond is slightly shortened (Δ = 0.028 Å) compared to N5—O16 and N5—O15, which lengthen due to coordination to Ce^3+^ and Cu^2+^ (Table 1 ▸). In complex **2**, the *L*^−^ and NO_3_^−^ ligands form six- and four-membered chelate metallacycles with the Gd^3+^ center, respectively. As in complex **1**, the O–S–N–P–O fragment in **2** is somewhat distorted [torsion angle O10—S2—N4—P2 = 26.6 (5)°]. The nitrate group forms an almost planar GdO_2_N fragment, as reflected by the small torsion angle ∠Gd1—O15—O16—N5)= 2.7 (4)° [the torsion angle for the cyclic bidentate *L*^−^ ligand is 6.70 (17)°]. For the O10/S2/N4/P2/O12 and O5/S1/N3/P1/O7 atom sets, the maximum deviations from the least-squares planes do not exceed 0.214 (4) and 0.440 (5) Å, respectively; the latter reflects a conformational change of the bridging O–S–N–P–O fragment due to the deviation of the phosphoryl group relative to the N3—S1 bond [torsion angle O7—P1—N3—S1 = −62.1 (5)°]. As in complex **1**, deprotonation of *L*^−^ in complex **2** results in similar increases in the P1—O7 and S1—O5 bond lengths by 0.007 and 0.008 Å, and decreases in the S1—N3 and P1—N3 bond lengths by 0.096 and 0.092 Å, respectively. The N5—O15, N5—O16, and N5—O17 bond lengths in the nitrate group coordinated to Gd^3+^ are 1.265 (5), 1.260 (5), and 1.214 (5) Å, respectively (Table 3 ▸). For comparison, in the complexes [Zn(Vanen)Ce(NO_3_)_3_] and [Zn(Vanen)Ce(NO_3_)_3_·CH_3_OH], the average N–O distances are *d*_avg_(N—O)_coord_ = 1.263 and 1.258 Å and *d*_avg_(N—O)_non-coord_ = 1.224 and 1.212 Å (Sui *et al.*, 2007 ▸). The lengthening of the N5—O15 and N5—O16 bonds compared to N5—O17 in complex **2** is caused by the coordination to Gd^3+^ (Table 2 ▸).

## 3. Supra­molecular features

**Figure 2:**
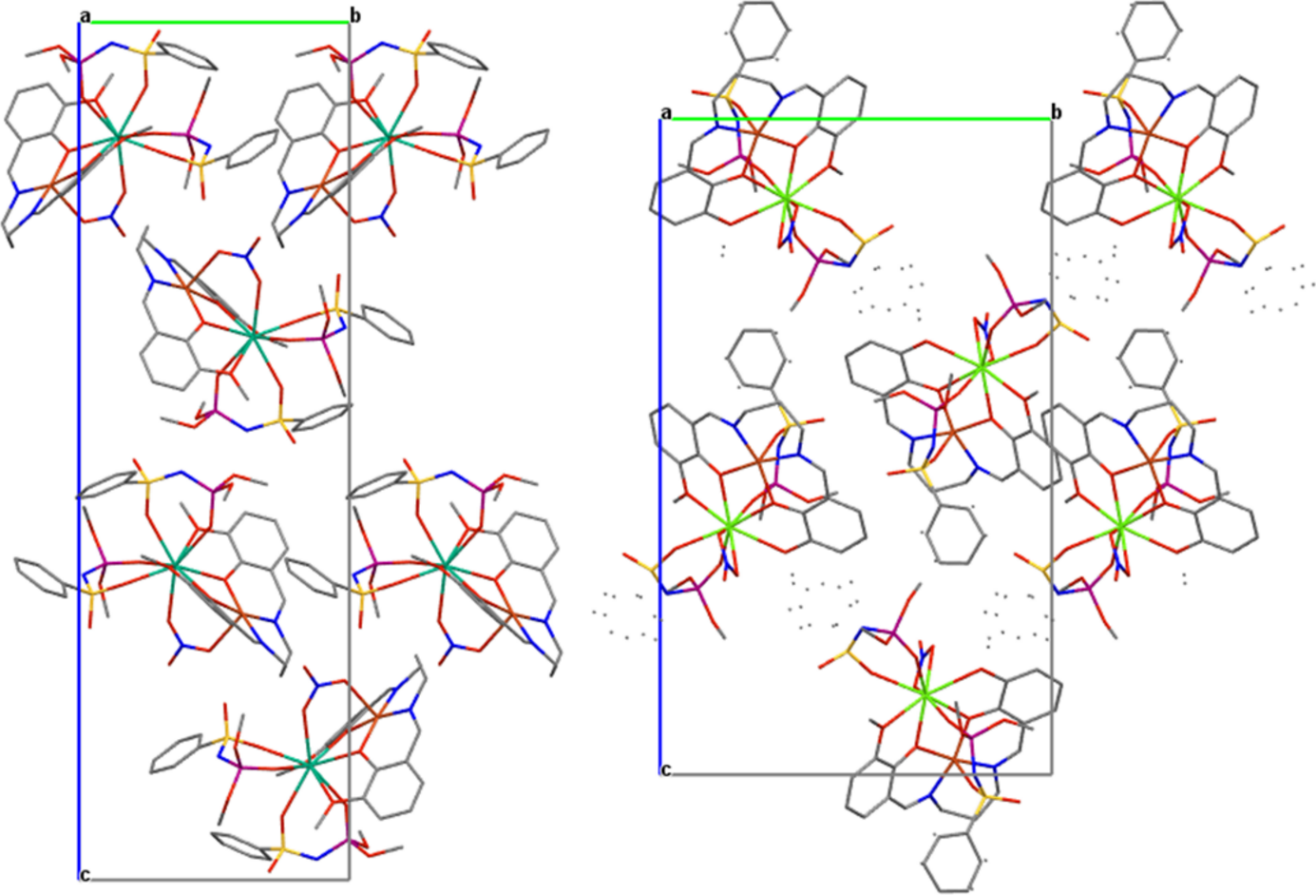
Crystal packings of **1** (left panel) and **2** (right panel) viewed down the *a* axis.

**Figure 3:**
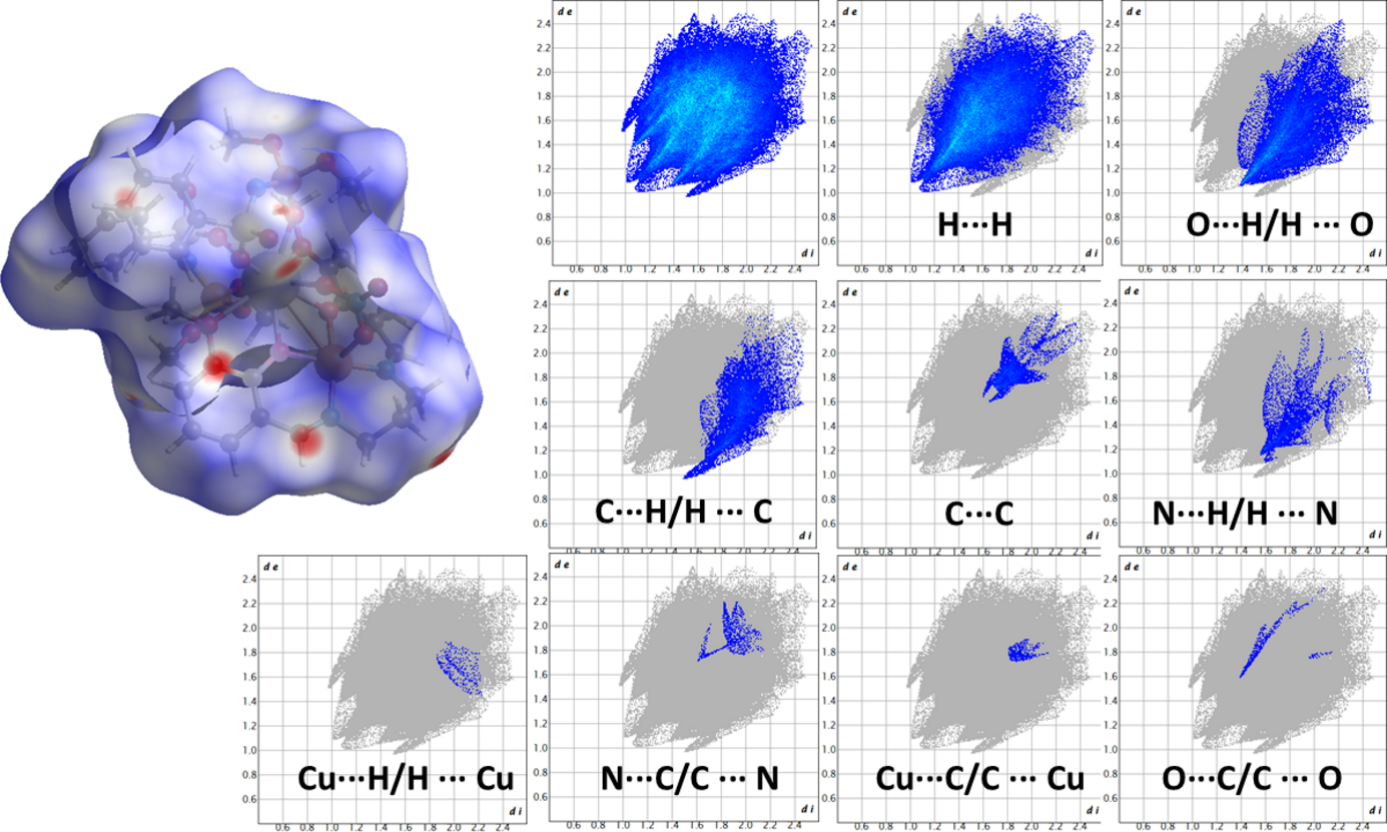
The Hirshfeld surface mapped over *d*_norm_ and the corresponding two-dimensional fingerprint plots showing all inter­molecular contacts and their delineated contributions (blue regions) in **1**. The parameters *d*_e_ and *d*_i_ represent the distances from a point on the Hirshfeld surface to the nearest external and inter­nal atoms, respectively. The relative contributions of individual contact types for the asymmetric unit of **1** are: H⋯H (45.6%), O⋯H/H⋯O (16.5%), C⋯H/H⋯C (7.3%), C⋯C (2.4%), N⋯H/H⋯N (2.2%), Cu⋯H/H⋯Cu (0.2%), N⋯C/C⋯N (0.5%), Cu⋯C/C⋯Cu (0.3%), and O⋯C/C⋯O (0.3%).

The crystal packing of both title compounds is illustrated in Fig. 2 ▸. For visualization of the main short inter­molecular inter­actions in the crystal packings for the asymmetric units of the title compounds, the Hirshfeld surface and its corresponding two-dimensional fingerprint plots (Spackman & Jayatilaka, 2009 ▸) were calculated for **1** and **2** using *CrystalExplorer17* (Turner *et al.*, 2017 ▸) (Figs. 3 ▸ and 4 ▸).

For complex **1**, the largest contribution arises from H⋯H contacts (45.6%), and the second most significant contribution originates from H⋯O/O⋯H inter­actions (16.5%), which appear as pronounced red areas on the *d*_norm_ surface. Smaller contributions are observed for C⋯H/H⋯C (7.3%), C⋯C (2.4%), and N⋯H/H⋯N (2.2%) inter­actions, while all other contact types (Cu⋯H/H⋯Cu, N⋯C/C⋯N, Cu⋯C/C⋯Cu, O⋯C/C⋯O, N⋯O/O⋯N) remain below 1%. In complex **2**, H⋯H inter­actions contribute an even larger fraction of the surface (51.4%). The proportion of H⋯O/O⋯H contacts decreases to 13.0%. The contributions from C⋯H/H⋯C (9.2%), N⋯H/H⋯N (2%) and C⋯C (1.7%) contacts remain comparable to those observed in **1**, while all other contacts (O⋯O, Cu⋯H/H⋯Cu, O⋯C/C⋯O, N⋯C/C⋯N and N⋯O/O⋯N) remain minor (<1%). Overall, the Hirshfeld surface analysis shows that both complexes are stabilized predominantly by dispersive H⋯H contacts, and the nature and balance of secondary inter­actions don’t differ. Complex **1** displays a stronger involvement of O⋯H contacts, whereas complex **2** features the larger H⋯H contact percentage.

## 4. Database survey

A search of the Cambridge Structural Database (CSD, version 5.43 with updates to November 2022; Groom *et al.*, 2016 ▸) was performed using a fragment-based query designed to identify heterometallic 3*d*–4*f* complexes containing a Schiff base ligand of the salen/vanillin type in combination with a phosphoryl-derived ligand. The query returned seven entries with refcodes NOFLAX, NOFLEB, NOFLIF, NOFLOL, NOFLUR, NOFMAY, and NOFMEC. All hits correspond to *Ln*–*M*′ complexes (*Ln* = La^3+^ or Eu^3+^; *M*′ = Ni^2+^ or Zn^2+^) reported by Amirkhanov *et al.* (2014 ▸). In these structures, the Schiff-base fragment acts as a tetra­dentate *O*,*N*,*O*,*N* chelator to the 3*d* metal, while the lanthanide ion is coordinated by two carbacyl­amido­phosphate ligands, together with an additional acetate group (NOFLEB, NOFLIF, NOFLOL, NOFLUR, NOFMAY, NOFMEC) or a nitrato ligand (NOFLAX). Several structures (NOFLUR and NOFMAY) also include methanol mol­ecules or solvent-separated species. Despite the similarity in coordination motifs, none of he retrieved structures exhibit a Cu–*Ln* framework comparable to the complexes described in this work. All database entries contain Zn^2+^ or Ni^2+^ as the 3*d* metal and incorporate a carbacyl­amido­phosphate ligand rather than the sulfonyl­amido­phosphate ligand present in the title compounds. Furthermore, no structures featuring a bridging nitrato ligand as observed in **1**, or a bridging phosphoryl ligand as observed in **2**, were identified.

## 5. Synthesis and crystallization

The azomethine-type ligand H_2_Vanpen was synthesized and identified using a modified procedure according to Costes *et al. (*1996), and the SAPh ligand H*L* as well as its salt Na*L* were obtained using the procedure described in Znovjyak *et al.* (2015 ▸).

For the synthesis of **1** and **2**, the salt Ce(NO_3_)_3_·4.62H_2_O (0.102 g, 0.25 mmol) or Gd(NO_3_)_3_·4.92H_2_O (0.108 g, 0.25 mmol) was dissolved in 2 mL of acetone and added to an acetone solution (3 mL) of Na*L* (0.144 g, 0.5 mmol). After 20 minutes, the precipitated NaNO_3_ was filtered off, and the resulting solution of Ce(*L*)_2_(NO_3_) or Gd(*L*)_2_(NO_3_) was added dropwise, under continuous stirring, to a hot chloro­form solution (5m*L*) of Cu(Vanpen) (0.101 g, 0.25 mmol). The resulting clear green solution was stirred for 15 minutes at room temperature and then left to evaporate in air until an oily residue formed. This residue was dissolved in an ethanol–chloro­form mixture (6:1) and left for crystallization by slow solvent evaporation at room temperature. After 4–6 days, fine green plate-like crystals formed for both complexes; these were filtered off, washed with cold acetone and diethyl ether, and dried in air. The complexes are soluble in DMF, DMSO, and aceto­nitrile, sparingly soluble in methanol, and insoluble in nonpolar aprotic solvents.

C_35_H_42_CeCuN_5_O_17_P_2_S_2_ (**1**): Yield 85%. IR (KBr), cm^−1^: 1640–1628 [ν(CN)], 1564 [(CC)], 1477 [ν_as_(NO_2_)], 1292 [ν_s_(NO_2_)], 1253–1232 [ν(CPh—O)], 1177 [ν(PO)].

C_35_H_42_CuGdN_5_O_17_P_2_S_2_ (**2**): Yield 65%. IR (KBr), cm^−1^: 1640–1625 [ν(CN)], 1566 [ν(CC)], 1473 ([_as_(NO_2_)], 1301 ([_s_(NO_2_)], 1240 [ν(CPh—O)], 1178 [ν(PO)].

## 6. Refinement

Crystal data, data collection and structure refinement details are summarized in Table 5 ▸. H atoms were placed in calculated positions and refined using a riding model, with C—H distances of 0.93–0.9 Å and with *U*_iso_(H) = 1.2*U*_eq_(C) (1.5*U*_eq_ for methyl H atoms).

## Supplementary Material

Crystal structure: contains datablock(s) 1, 2. DOI: 10.1107/S2056989026003002/ee2028sup1.cif

Structure factors: contains datablock(s) 1. DOI: 10.1107/S2056989026003002/ee20281sup2.hkl

Structure factors: contains datablock(s) 2. DOI: 10.1107/S2056989026003002/ee20282sup3.hkl

CCDC references: 2539664, 2539663

Additional supporting information:  crystallographic information; 3D view; checkCIF report

## Figures and Tables

**Figure 4:**
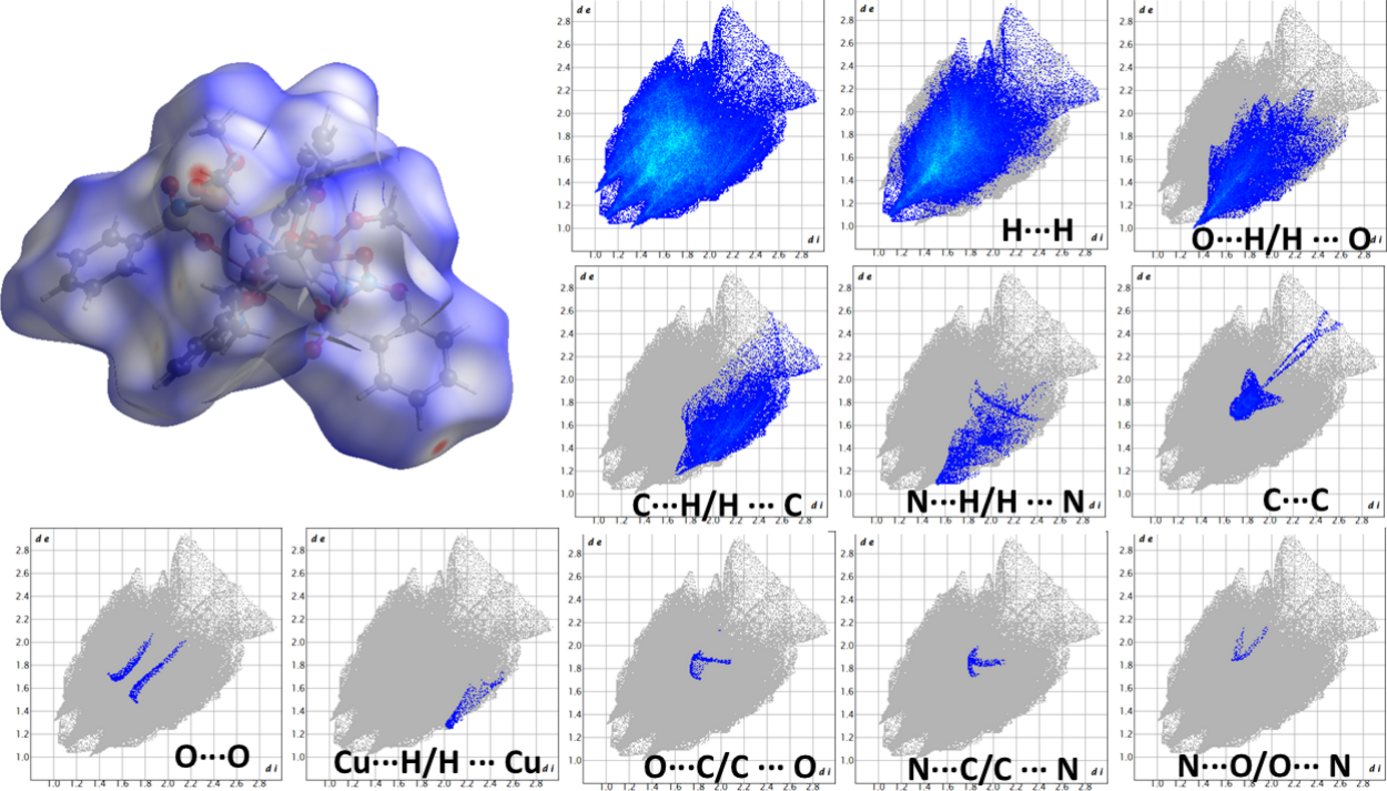
The Hirshfeld surface mapped over d_norm_ and the corresponding two-dimensional fingerprint plots showing all inter­molecular contacts and their delineated contributions (blue regions) in **2**. The parameters *d*_e_ and *d*_i_ represent the distances from a point on the Hirshfeld surface to the nearest external and inter­nal atoms, respectively. The relative contributions of individual contact types for the asymmetric unit of **2** are: H⋯H (51.4%), O⋯H/H⋯O (13%), C⋯H/H⋯C (9.2%), N⋯H/H⋯N (2.0%), C⋯C (1.7%), O⋯O (0.4%), Cu⋯H/H⋯Cu (0.2%), O⋯C/C⋯O (0.2%), N⋯C/C⋯N (0.1%), and N⋯O/O⋯N (0.1%).

**Table 1:** Selected geometric parameters (Å, °) for **1**[Chem scheme1]

Ce1—O1	2.716 (3)	S1—O5	1.465 (3)
Ce1—O2	2.469 (3)	S1—O6	1.438 (3)
Ce1—O3	2.448 (3)	S1—N3	1.548 (4)
Ce1—O4	2.661 (3)	S2—O10	1.474 (3)
Ce1—O5	2.506 (3)	S2—O11	1.434 (4)
Ce1—O7	2.468 (3)	S2—N4	1.533 (4)
Ce1—O10	2.496 (3)	P1—O7	1.486 (3)
Ce1—O12	2.437 (3)	P1—N3	1.586 (4)
Ce1—O16	2.573 (3)	P2—O12	1.480 (3)
Cu1—O2	1.966 (3)	P2—N4	1.589 (4)
Cu1—O3	1.979 (3)	O15—N5	1.239 (5)
Cu1—O15	2.329 (3)	O16—N5	1.253 (5)
Cu1—N1	1.964 (4)	O17—N5	1.218 (5)
Cu1—N2	1.983 (4)		
			
O2—Ce1—O1	60.44 (9)	O2—Cu1—O15	95.26 (13)
O2—Ce1—O16	75.19 (10)	O2—Cu1—N2	170.36 (14)
O3—Ce1—O2	63.72 (9)	O3—Cu1—O15	91.17 (13)
O3—Ce1—O4	61.22 (9)	O3—Cu1—N2	89.90 (14)
O4—Ce1—O1	139.91 (10)	N1—Cu1—O2	91.93 (14)
O5—Ce1—O16	142.48 (10)	N1—Cu1—O3	169.67 (15)
O7—Ce1—O5	69.96 (10)	N1—Cu1—O15	97.89 (15)
O12—Ce1—O10	72.46 (10)	N1—Cu1—N2	94.94 (16)
O16—Ce1—O4	108.01 (11)	N2—Cu1—O15	90.50 (15)
O2—Cu1—O3	82.27 (11)	O15—N5—O16	119.5 (4)

**Table 2:** Selected geometric parameters (Å, °) for **2**[Chem scheme1]

Gd1—O1	2.570 (3)	S1—O5	1.431 (3)
Gd1—O2	2.354 (3)	S1—O6	1.433 (4)
Gd1—O3	2.376 (3)	S1—N3	1.546 (4)
Gd1—O4	2.578 (3)	S2—O10	1.461 (3)
Gd1—O7	2.284 (3)	S2—O11	1.428 (4)
Gd1—O10	2.489 (3)	S2—N4	1.552 (4)
Gd1—O12	2.311 (3)	P1—O7	1.463 (3)
Gd1—O15	2.475 (3)	P1—N3	1.570 (4)
Gd1—O16	2.508 (3)	P2—O12	1.471 (3)
Cu1—O2	1.965 (3)	P2—N4	1.568 (4)
Cu1—O3	1.956 (3)	O15—N5	1.265 (5)
Cu1—O5	2.372 (3)	O16—N5	1.260 (5)
Cu1—N1	1.965 (4)	O17—N5	1.214 (5)
Cu1—N2	1.986 (4)		
			
O2—Gd1—O1	63.41 (9)	O2—Cu1—N2	172.60 (13)
O2—Gd1—O3	64.67 (9)	O3—Cu1—O2	80.36 (11)
O3—Gd1—O4	63.65 (9)	O3—Cu1—O5	91.95 (13)
O7—Gd1—O1	71.16 (12)	O3—Cu1—N1	165.91 (15)
O7—Gd1—O2	84.85 (11)	O3—Cu1—N2	92.73 (13)
O7—Gd1—O12	80.96 (11)	N1—Cu1—O2	90.41 (14)
O7—Gd1—O16	152.88 (11)	N1—Cu1—O5	98.53 (16)
O12—Gd1—O10	72.70 (10)	N1—Cu1—N2	95.88 (15)
O15—Gd1—O16	51.24 (11)	N2—Cu1—O5	94.06 (14)
O2—Cu1—O5	88.84 (13)	O16—N5—O15	117.2 (4)

**Table 3:** Selected inter­planar and dihedral angles in **1** (°)

Cu1O3O2–Ce1O3O2	17.49 (8)
Cu1N2O3–Cu1N1O2	10.88 (14)
Cu1N1N2–Cu1N2O3	9.14 (12)
Cu1N1N2–Cu1N1O2	6.78 (13)
Cu1N1N2–Cu1O3O2	10.74 (11)
Cu1O3Ce1–Ce1O3C17	22.1 (2)
Cu1O2Ce1–Ce1O2C1	9.9 (2)

**Table 4:** Selected inter­planar and dihedral angles in **2** (°)

Cu1O2O3–Gd1O2O3	4.00 (9)
Cu1N1O2–Cu1N2O3	11.44 (12)
Cu1N1N2–Cu1N1O2	3.91 (12)
Cu1N1N2–Cu1N2O3	11.17 (13)
Cu1N1N2–Cu1O2O3	11.35 (11)
Cu1O2Gd1–Gd1O2C1	19.9 (2)
Cu1O3Gd1–Gd1O3C17	13.1 (2)

**Table 5:** Experimental details

	**1**	**2**
Crystal data		
Chemical formula	[CeCu(C_19_H_20_N_2_O_4_)(C_8_H_11_NO_5_PS)_2_(NO_3_)]	[CuGd(C_19_H_20_N_2_O_4_)(C_8_H_11_NO_5_PS)_2_(NO_3_)]
*M*_r_	1134.45	1151.58
Crystal system, space group	Monoclinic, *P*2_1_/*n*	Monoclinic, *P*2_1_/*n*
Temperature (K)	293	293
*a*, *b*, *c* (Å)	10.0242 (3), 11.5239 (3), 36.6090 (9)	9.9250 (2), 16.3468 (4), 27.4479 (6)
β (°)	93.432 (2)	93.978 (2)
*V* (Å^3^)	4221.39 (18)	4442.47 (17)
*Z*	4	4
Radiation type	Mo *K*α	Mo *K*α
μ (mm^−1^)	1.82	2.20
Crystal size (mm)	0.4 × 0.2 × 0.1	0.3 × 0.2 × 0.1

Data collection		
Diffractometer	Xcalibur, Sapphire3	Bruker APEXII CCD
Absorption correction	Multi-scan (*CrysAlis PRO*; Rigaku OD, 2024 ▸)	Numerical (*CrysAlis PRO*; Rigaku OD, 2024 ▸)
*T*_min_, *T*_max_	0.740, 1.000	0.111, 0.342
No. of measured, independent and observed [*I* > 2σ(*I*)] reflections	23364, 8286, 6601	38125, 8728, 7249
*R*_int_	0.045	0.038
(sin θ/λ)_max_ (Å^−1^)	0.617	0.617

Refinement		
*R*[*F*^2^ > 2σ(*F*^2^)], *wR*(*F*^2^), *S*	0.043, 0.093, 1.07	0.038, 0.091, 1.07
No. of reflections	8286	8728
No. of parameters	574	550
No. of restraints	0	60
H-atom treatment	H-atom parameters constrained	H-atom parameters constrained
Δρ_max_, Δρ_min_ (e Å^−3^)	0.92, −0.48	0.91, −0.51

Computer programs: *CrysAlis PRO* (Rigaku OD, 2024 ▸), *SHELXT* (Sheldrick, 2015*a* ▸), *SHELXL* (Sheldrick, 2015*b* ▸) and *OLEX2* (Dolomanov *et al.*, 2009 ▸).

## supplementary crystallographic information

### Bis[dimethyl (phenylsulfonyl)amidophosphato]-µ-nitrato-(µ-6,6'-{(1E,1'E)-[propane-1,3-diylbis(azanylylidene)]bis(methanylylidene)}bis(2-methoxyphenolato))cerium(III)copper(II) (1) Crystal data

**Table:**

[CeCu(C_19_H_20_N_2_O_4_)(C_8_H_11_NO_5_PS)_2_(NO_3_)]	*F*(000) = 2288
*M**_r_* = 1134.45	*D*_x_ = 1.785 Mg m^−^^3^
Monoclinic, *P*2_1_/*n*	Mo *K*α radiation, λ = 0.71073 Å
*a* = 10.0242 (3) Å	Cell parameters from 7551 reflections
*b* = 11.5239 (3) Å	θ = 3.0–29.0°
*c* = 36.6090 (9) Å	µ = 1.82 mm^−^^1^
β = 93.432 (2)°	*T* = 293 K
*V* = 4221.39 (18) Å^3^	Block, colourless
*Z* = 4	0.4 × 0.2 × 0.1 mm

### Bis[dimethyl (phenylsulfonyl)amidophosphato]-µ-nitrato-(µ-6,6'-{(1E,1'E)-[propane-1,3-diylbis(azanylylidene)]bis(methanylylidene)}bis(2-methoxyphenolato))cerium(III)copper(II) (1) Data collection

**Table:**

Xcalibur, Sapphire3 diffractometer	6601 reflections with *I* > 2σ(*I*)
Detector resolution: 16.1827 pixels mm^-1^	*R*_int_ = 0.045
ω scans	θ_max_ = 26.0°, θ_min_ = 3.0°
Absorption correction: multi-scan (CrysAlisPro; Rigaku OD, 2024)	*h* = −12→11
*T*_min_ = 0.740, *T*_max_ = 1.000	*k* = −13→14
23364 measured reflections	*l* = −45→44
8286 independent reflections	

### Bis[dimethyl (phenylsulfonyl)amidophosphato]-µ-nitrato-(µ-6,6'-{(1E,1'E)-[propane-1,3-diylbis(azanylylidene)]bis(methanylylidene)}bis(2-methoxyphenolato))cerium(III)copper(II) (1) Refinement

**Table:**

Refinement on *F*^2^	Primary atom site location: dual
Least-squares matrix: full	Hydrogen site location: inferred from neighbouring sites
*R*[*F*^2^ > 2σ(*F*^2^)] = 0.043	H-atom parameters constrained
*wR*(*F*^2^) = 0.093	*w* = 1/[σ^2^(*F*_o_^2^) + (0.0326*P*)^2^ + 3.3878*P*] where *P* = (*F*_o_^2^ + 2*F*_c_^2^)/3
*S* = 1.07	(Δ/σ)_max_ = 0.001
8286 reflections	Δρ_max_ = 0.92 e Å^−^^3^
574 parameters	Δρ_min_ = −0.48 e Å^−^^3^
0 restraints	

### Bis[dimethyl (phenylsulfonyl)amidophosphato]-µ-nitrato-(µ-6,6'-{(1E,1'E)-[propane-1,3-diylbis(azanylylidene)]bis(methanylylidene)}bis(2-methoxyphenolato))cerium(III)copper(II) (1) Special details

**Table:**

Geometry. All esds (except the esd in the dihedral angle between two l.s. planes) are estimated using the full covariance matrix. The cell esds are taken into account individually in the estimation of esds in distances, angles and torsion angles; correlations between esds in cell parameters are only used when they are defined by crystal symmetry. An approximate (isotropic) treatment of cell esds is used for estimating esds involving l.s. planes.

### Bis[dimethyl (phenylsulfonyl)amidophosphato]-µ-nitrato-(µ-6,6'-{(1E,1'E)-[propane-1,3-diylbis(azanylylidene)]bis(methanylylidene)}bis(2-methoxyphenolato))cerium(III)copper(II) (1) Fractional atomic coordinates and isotropic or equivalent isotropic displacement parameters (Å^2^)

**Table:**

	*x*	*y*	*z*	*U*_iso_*/*U*_eq_	
Ce1	0.87864 (2)	0.14469 (2)	0.13348 (2)	0.02835 (8)	
Cu1	0.88630 (5)	−0.10430 (4)	0.18988 (2)	0.03142 (13)	
S1	0.77738 (13)	0.24400 (11)	0.03617 (3)	0.0414 (3)	
S2	0.86894 (12)	0.45122 (10)	0.16643 (3)	0.0377 (3)	
P1	0.74181 (13)	0.00392 (11)	0.04742 (3)	0.0388 (3)	
P2	1.10226 (12)	0.40074 (11)	0.13109 (4)	0.0403 (3)	
O1	0.6093 (3)	0.1664 (3)	0.13539 (8)	0.0381 (7)	
O2	0.7546 (3)	0.0124 (2)	0.17226 (8)	0.0320 (7)	
O3	0.9884 (3)	−0.0393 (2)	0.15003 (8)	0.0319 (7)	
O4	1.0603 (3)	0.0607 (3)	0.09040 (8)	0.0380 (7)	
O5	0.8334 (3)	0.2493 (3)	0.07402 (8)	0.0414 (8)	
O6	0.8630 (4)	0.2958 (3)	0.01058 (9)	0.0572 (10)	
O7	0.7713 (3)	0.0123 (3)	0.08758 (8)	0.0370 (7)	
O8	0.6100 (3)	−0.0667 (3)	0.03831 (10)	0.0562 (10)	
O9	0.8448 (3)	−0.0735 (3)	0.02785 (9)	0.0519 (9)	
O10	0.8084 (3)	0.3390 (2)	0.15549 (9)	0.0396 (8)	
O11	0.8954 (4)	0.4605 (3)	0.20525 (10)	0.0595 (10)	
O12	1.0677 (3)	0.2759 (3)	0.12934 (9)	0.0403 (8)	
O13	1.1491 (4)	0.4491 (3)	0.09399 (10)	0.0558 (10)	
O14	1.2362 (3)	0.4227 (3)	0.15447 (11)	0.0565 (10)	
O15	0.9969 (4)	0.0173 (3)	0.23257 (10)	0.0619 (11)	
O16	0.9904 (4)	0.1689 (3)	0.19815 (9)	0.0507 (9)	
O17	1.1122 (4)	0.1663 (3)	0.24829 (10)	0.0621 (10)	
N1	0.7598 (4)	−0.1740 (3)	0.22246 (10)	0.0423 (10)	
N2	1.0255 (4)	−0.2250 (3)	0.19887 (10)	0.0362 (9)	
N3	0.7331 (5)	0.1203 (4)	0.02425 (11)	0.0518 (11)	
N4	0.9881 (4)	0.4840 (3)	0.14418 (13)	0.0504 (11)	
N5	1.0345 (4)	0.1175 (3)	0.22652 (11)	0.0384 (9)	
C1	0.6235 (4)	0.0086 (4)	0.17587 (11)	0.0300 (9)	
C2	0.5401 (4)	0.0878 (4)	0.15569 (12)	0.0338 (10)	
C3	0.4043 (5)	0.0865 (4)	0.15759 (13)	0.0417 (12)	
H3	0.351391	0.136925	0.143154	0.050*	
C4	0.3451 (5)	0.0093 (5)	0.18123 (15)	0.0490 (13)	
H4	0.252788	0.008630	0.182743	0.059*	
C5	0.4228 (5)	−0.0645 (4)	0.20191 (15)	0.0481 (13)	
H5	0.383106	−0.114540	0.217984	0.058*	
C6	0.5620 (5)	−0.0671 (4)	0.19960 (13)	0.0380 (11)	
C7	0.6372 (5)	−0.1494 (4)	0.22253 (13)	0.0440 (12)	
H7	0.588985	−0.189321	0.239454	0.053*	
C8	0.8071 (6)	−0.2645 (5)	0.24916 (16)	0.0667 (17)	
H8A	0.751992	−0.262908	0.270060	0.080*	
H8B	0.797437	−0.340350	0.237811	0.080*	
C9	0.9480 (6)	−0.2463 (5)	0.26175 (14)	0.0613 (16)	
H9A	0.966196	−0.288970	0.284348	0.074*	
H9B	0.961704	−0.164595	0.267074	0.074*	
C10	1.0458 (5)	−0.2837 (5)	0.23466 (13)	0.0500 (13)	
H10A	1.135707	−0.267786	0.244685	0.060*	
H10B	1.037988	−0.366871	0.230999	0.060*	
C11	1.1014 (4)	−0.2601 (4)	0.17435 (13)	0.0384 (11)	
H11	1.154275	−0.324455	0.180303	0.046*	
C12	1.1146 (4)	−0.2112 (4)	0.13847 (13)	0.0353 (10)	
C13	1.1920 (5)	−0.2713 (4)	0.11378 (15)	0.0459 (13)	
H13	1.226289	−0.343965	0.120222	0.055*	
C14	1.2175 (5)	−0.2255 (4)	0.08079 (15)	0.0505 (14)	
H14	1.264410	−0.268459	0.064299	0.061*	
C15	1.1735 (5)	−0.1147 (4)	0.07171 (14)	0.0428 (12)	
H15	1.192221	−0.082655	0.049279	0.051*	
C16	1.1023 (4)	−0.0523 (4)	0.09586 (12)	0.0309 (10)	
C17	1.0648 (4)	−0.1016 (3)	0.12891 (11)	0.0279 (9)	
C18	0.5341 (5)	0.2661 (4)	0.12241 (14)	0.0521 (14)	
H18A	0.470885	0.243101	0.103030	0.078*	
H18B	0.593940	0.323008	0.113402	0.078*	
H18C	0.487447	0.298711	0.142120	0.078*	
C19	1.1168 (6)	0.1222 (5)	0.06072 (14)	0.0572 (15)	
H19A	1.212426	0.115393	0.062895	0.086*	
H19B	1.092207	0.202593	0.061674	0.086*	
H19C	1.083546	0.089532	0.037836	0.086*	
C20	0.6317 (5)	0.3310 (4)	0.03635 (13)	0.0443 (12)	
C21	0.6465 (6)	0.4406 (5)	0.05165 (15)	0.0562 (14)	
H21	0.730532	0.467383	0.059877	0.067*	
C22	0.5355 (7)	0.5094 (5)	0.05453 (18)	0.0715 (18)	
H22	0.544674	0.583416	0.064475	0.086*	
C23	0.4137 (7)	0.4700 (6)	0.04302 (19)	0.0779 (19)	
H23	0.339329	0.516863	0.045674	0.093*	
C24	0.3969 (6)	0.3628 (6)	0.02754 (19)	0.0770 (19)	
H24	0.312088	0.337796	0.019329	0.092*	
C25	0.5075 (6)	0.2907 (5)	0.02409 (16)	0.0635 (16)	
H25	0.497552	0.217238	0.013761	0.076*	
C26	0.4914 (5)	−0.0386 (6)	0.05638 (18)	0.0728 (18)	
H26A	0.416782	−0.079799	0.044971	0.109*	
H26B	0.475149	0.043410	0.054590	0.109*	
H26C	0.502600	−0.060395	0.081680	0.109*	
C27	0.8640 (6)	−0.1937 (5)	0.03859 (16)	0.0603 (15)	
H27A	0.931400	−0.227936	0.024455	0.090*	
H27B	0.781577	−0.235220	0.034309	0.090*	
H27C	0.891842	−0.197410	0.064112	0.090*	
C28	0.7465 (5)	0.5583 (4)	0.15445 (13)	0.0376 (11)	
C29	0.7738 (5)	0.6511 (4)	0.13249 (13)	0.0437 (12)	
H29	0.856676	0.658182	0.122579	0.052*	
C30	0.6749 (6)	0.7342 (4)	0.12539 (15)	0.0522 (13)	
H30	0.692349	0.798217	0.110964	0.063*	
C31	0.5525 (5)	0.7226 (5)	0.13940 (15)	0.0530 (14)	
H31	0.486150	0.777273	0.133909	0.064*	
C32	0.5274 (6)	0.6301 (5)	0.16161 (17)	0.0612 (16)	
H32	0.444413	0.622678	0.171469	0.073*	
C33	0.6246 (5)	0.5488 (4)	0.16920 (16)	0.0554 (14)	
H33	0.607816	0.486618	0.184468	0.066*	
C34	1.0554 (6)	0.4820 (5)	0.06485 (16)	0.0701 (18)	
H34A	1.100035	0.526865	0.047172	0.105*	
H34B	0.985372	0.527534	0.074472	0.105*	
H34C	1.017749	0.413595	0.053345	0.105*	
C35	1.2416 (6)	0.3927 (5)	0.19294 (17)	0.0698 (17)	
H35A	1.242568	0.309754	0.195533	0.105*	
H35B	1.164547	0.423679	0.203893	0.105*	
H35C	1.321110	0.424628	0.204902	0.105*	

### Bis[dimethyl (phenylsulfonyl)amidophosphato]-µ-nitrato-(µ-6,6'-{(1E,1'E)-[propane-1,3-diylbis(azanylylidene)]bis(methanylylidene)}bis(2-methoxyphenolato))cerium(III)copper(II) (1) Atomic displacement parameters (Å^2^)

**Table:**

	*U*^11^	*U*^22^	*U*^33^	*U*^12^	*U*^13^	*U*^23^
Ce1	0.03128 (14)	0.02753 (14)	0.02663 (13)	0.00147 (10)	0.00507 (9)	0.00139 (11)
Cu1	0.0362 (3)	0.0314 (3)	0.0272 (3)	0.0018 (2)	0.0056 (2)	0.0041 (2)
S1	0.0492 (7)	0.0449 (7)	0.0300 (6)	0.0039 (5)	0.0022 (5)	0.0052 (5)
S2	0.0411 (7)	0.0298 (6)	0.0424 (7)	0.0025 (5)	0.0050 (5)	−0.0004 (5)
P1	0.0413 (7)	0.0423 (7)	0.0329 (7)	0.0000 (5)	0.0032 (5)	−0.0065 (6)
P2	0.0341 (7)	0.0351 (7)	0.0525 (8)	−0.0028 (5)	0.0078 (6)	0.0042 (6)
O1	0.0355 (17)	0.0414 (18)	0.0379 (18)	0.0081 (13)	0.0054 (14)	0.0062 (15)
O2	0.0309 (16)	0.0364 (17)	0.0291 (16)	0.0029 (12)	0.0062 (13)	0.0036 (14)
O3	0.0368 (17)	0.0317 (16)	0.0280 (16)	0.0065 (13)	0.0090 (13)	0.0032 (13)
O4	0.0442 (19)	0.0368 (18)	0.0348 (17)	0.0051 (14)	0.0176 (14)	0.0074 (14)
O5	0.050 (2)	0.0417 (19)	0.0317 (17)	0.0006 (15)	−0.0052 (15)	0.0048 (15)
O6	0.064 (2)	0.066 (2)	0.043 (2)	0.0009 (19)	0.0177 (18)	0.0112 (19)
O7	0.0437 (19)	0.0395 (18)	0.0277 (16)	−0.0048 (14)	0.0007 (14)	−0.0017 (14)
O8	0.045 (2)	0.068 (2)	0.055 (2)	−0.0077 (17)	0.0016 (17)	−0.013 (2)
O9	0.059 (2)	0.050 (2)	0.048 (2)	0.0051 (17)	0.0154 (17)	−0.0062 (18)
O10	0.0384 (18)	0.0270 (16)	0.055 (2)	0.0002 (13)	0.0155 (15)	−0.0018 (15)
O11	0.078 (3)	0.053 (2)	0.046 (2)	0.0126 (19)	−0.0091 (19)	−0.0017 (19)
O12	0.0383 (18)	0.0340 (17)	0.050 (2)	−0.0014 (13)	0.0096 (15)	0.0002 (15)
O13	0.052 (2)	0.056 (2)	0.062 (2)	−0.0043 (17)	0.0182 (19)	0.016 (2)
O14	0.037 (2)	0.055 (2)	0.078 (3)	−0.0083 (16)	0.0018 (18)	0.000 (2)
O15	0.097 (3)	0.039 (2)	0.047 (2)	−0.0231 (19)	−0.013 (2)	0.0057 (18)
O16	0.069 (2)	0.042 (2)	0.039 (2)	−0.0023 (17)	−0.0099 (17)	0.0049 (17)
O17	0.063 (3)	0.063 (2)	0.058 (2)	−0.0130 (19)	−0.018 (2)	−0.008 (2)
N1	0.048 (3)	0.045 (2)	0.035 (2)	−0.0005 (18)	0.0092 (18)	0.0089 (19)
N2	0.039 (2)	0.031 (2)	0.038 (2)	0.0020 (16)	−0.0024 (18)	0.0052 (18)
N3	0.079 (3)	0.044 (3)	0.030 (2)	0.000 (2)	−0.006 (2)	0.0013 (19)
N4	0.041 (2)	0.034 (2)	0.079 (3)	−0.0056 (17)	0.019 (2)	0.004 (2)
N5	0.041 (2)	0.039 (2)	0.035 (2)	0.0035 (18)	0.0012 (18)	−0.0074 (19)
C1	0.034 (2)	0.034 (2)	0.023 (2)	−0.0025 (18)	0.0061 (18)	−0.0072 (19)
C2	0.035 (2)	0.039 (3)	0.029 (2)	−0.0006 (19)	0.0065 (19)	−0.012 (2)
C3	0.034 (3)	0.051 (3)	0.040 (3)	0.002 (2)	−0.002 (2)	−0.012 (2)
C4	0.029 (3)	0.061 (3)	0.057 (3)	−0.010 (2)	0.008 (2)	−0.018 (3)
C5	0.043 (3)	0.049 (3)	0.053 (3)	−0.013 (2)	0.017 (2)	−0.005 (3)
C6	0.037 (3)	0.039 (3)	0.039 (3)	−0.008 (2)	0.009 (2)	−0.008 (2)
C7	0.054 (3)	0.042 (3)	0.038 (3)	−0.008 (2)	0.017 (2)	0.002 (2)
C8	0.073 (4)	0.072 (4)	0.056 (4)	−0.005 (3)	0.015 (3)	0.038 (3)
C9	0.088 (5)	0.061 (4)	0.034 (3)	−0.007 (3)	−0.002 (3)	0.017 (3)
C10	0.055 (3)	0.053 (3)	0.041 (3)	0.008 (2)	−0.006 (2)	0.017 (3)
C11	0.036 (3)	0.024 (2)	0.055 (3)	0.0027 (19)	0.000 (2)	0.004 (2)
C12	0.031 (2)	0.029 (2)	0.045 (3)	−0.0019 (18)	0.003 (2)	−0.004 (2)
C13	0.039 (3)	0.031 (3)	0.070 (4)	0.001 (2)	0.016 (3)	−0.004 (3)
C14	0.051 (3)	0.043 (3)	0.060 (4)	0.001 (2)	0.025 (3)	−0.018 (3)
C15	0.043 (3)	0.043 (3)	0.044 (3)	0.000 (2)	0.017 (2)	−0.006 (2)
C16	0.028 (2)	0.030 (2)	0.035 (2)	0.0020 (17)	0.0055 (19)	−0.004 (2)
C17	0.025 (2)	0.028 (2)	0.030 (2)	−0.0030 (17)	0.0001 (17)	−0.0046 (19)
C18	0.046 (3)	0.061 (3)	0.050 (3)	0.015 (2)	0.004 (2)	0.017 (3)
C19	0.067 (4)	0.055 (3)	0.053 (3)	0.013 (3)	0.031 (3)	0.021 (3)
C20	0.050 (3)	0.048 (3)	0.035 (3)	0.006 (2)	0.002 (2)	0.008 (2)
C21	0.062 (4)	0.050 (3)	0.056 (4)	0.001 (3)	−0.002 (3)	−0.002 (3)
C22	0.070 (4)	0.058 (4)	0.086 (5)	0.016 (3)	0.010 (4)	0.000 (4)
C23	0.068 (5)	0.081 (5)	0.085 (5)	0.023 (4)	0.011 (4)	−0.002 (4)
C24	0.051 (4)	0.094 (5)	0.084 (5)	0.004 (3)	−0.008 (3)	0.006 (4)
C25	0.065 (4)	0.065 (4)	0.059 (4)	0.001 (3)	−0.007 (3)	0.001 (3)
C26	0.038 (3)	0.097 (5)	0.084 (5)	−0.011 (3)	0.010 (3)	0.002 (4)
C27	0.058 (4)	0.053 (3)	0.071 (4)	0.005 (3)	0.007 (3)	−0.009 (3)
C28	0.040 (3)	0.030 (2)	0.043 (3)	0.0017 (19)	0.005 (2)	−0.005 (2)
C29	0.043 (3)	0.042 (3)	0.047 (3)	0.000 (2)	0.006 (2)	0.004 (2)
C30	0.061 (4)	0.044 (3)	0.052 (3)	0.004 (3)	0.000 (3)	0.007 (3)
C31	0.051 (3)	0.049 (3)	0.058 (3)	0.012 (2)	−0.002 (3)	−0.005 (3)
C32	0.047 (3)	0.055 (4)	0.083 (4)	0.006 (3)	0.022 (3)	−0.003 (3)
C33	0.051 (3)	0.041 (3)	0.076 (4)	0.001 (2)	0.022 (3)	0.007 (3)
C34	0.078 (4)	0.081 (4)	0.051 (4)	−0.026 (3)	0.002 (3)	0.005 (3)
C35	0.056 (4)	0.075 (4)	0.077 (4)	−0.005 (3)	−0.015 (3)	0.013 (4)

### Bis[dimethyl (phenylsulfonyl)amidophosphato]-µ-nitrato-(µ-6,6'-{(1E,1'E)-[propane-1,3-diylbis(azanylylidene)]bis(methanylylidene)}bis(2-methoxyphenolato))cerium(III)copper(II) (1) Geometric parameters (Å, º)

**Table:**

Ce1—Cu1	3.5334 (6)	C8—H8A	0.9700
Ce1—O1	2.716 (3)	C8—H8B	0.9700
Ce1—O2	2.469 (3)	C8—C9	1.475 (8)
Ce1—O3	2.448 (3)	C9—H9A	0.9700
Ce1—O4	2.661 (3)	C9—H9B	0.9700
Ce1—O5	2.506 (3)	C9—C10	1.499 (7)
Ce1—O7	2.468 (3)	C10—H10A	0.9700
Ce1—O10	2.496 (3)	C10—H10B	0.9700
Ce1—O12	2.437 (3)	C11—H11	0.9300
Ce1—O16	2.573 (3)	C11—C12	1.442 (6)
Cu1—O2	1.966 (3)	C12—C13	1.408 (6)
Cu1—O3	1.979 (3)	C12—C17	1.396 (6)
Cu1—O15	2.329 (3)	C13—H13	0.9300
Cu1—N1	1.964 (4)	C13—C14	1.356 (7)
Cu1—N2	1.983 (4)	C14—H14	0.9300
S1—O5	1.465 (3)	C14—C15	1.385 (7)
S1—O6	1.438 (3)	C15—H15	0.9300
S1—N3	1.548 (4)	C15—C16	1.373 (6)
S1—C20	1.771 (5)	C16—C17	1.408 (6)
S2—O10	1.474 (3)	C18—H18A	0.9600
S2—O11	1.434 (4)	C18—H18B	0.9600
S2—N4	1.533 (4)	C18—H18C	0.9600
S2—C28	1.777 (5)	C19—H19A	0.9600
P1—O7	1.486 (3)	C19—H19B	0.9600
P1—O8	1.571 (4)	C19—H19C	0.9600
P1—O9	1.570 (3)	C20—C21	1.387 (7)
P1—N3	1.586 (4)	C20—C25	1.379 (7)
P2—O12	1.480 (3)	C21—H21	0.9300
P2—O13	1.566 (4)	C21—C22	1.375 (8)
P2—O14	1.569 (4)	C22—H22	0.9300
P2—N4	1.589 (4)	C22—C23	1.347 (8)
O1—C2	1.384 (5)	C23—H23	0.9300
O1—C18	1.439 (5)	C23—C24	1.366 (9)
O2—C1	1.330 (5)	C24—H24	0.9300
O3—C17	1.331 (5)	C24—C25	1.397 (8)
O4—C16	1.380 (5)	C25—H25	0.9300
O4—C19	1.441 (5)	C26—H26A	0.9600
O8—C26	1.432 (6)	C26—H26B	0.9600
O9—C27	1.449 (6)	C26—H26C	0.9600
O13—C34	1.430 (6)	C27—H27A	0.9600
O14—C35	1.448 (7)	C27—H27B	0.9600
O15—N5	1.239 (5)	C27—H27C	0.9600
O16—N5	1.253 (5)	C28—C29	1.375 (6)
O17—N5	1.218 (5)	C28—C33	1.370 (6)
N1—C7	1.260 (6)	C29—H29	0.9300
N1—C8	1.488 (6)	C29—C30	1.392 (7)
N2—C10	1.478 (6)	C30—H30	0.9300
N2—C11	1.278 (6)	C30—C31	1.364 (7)
C1—C2	1.416 (6)	C31—H31	0.9300
C1—C6	1.400 (6)	C31—C32	1.373 (7)
C2—C3	1.366 (6)	C32—H32	0.9300
C3—H3	0.9300	C32—C33	1.368 (7)
C3—C4	1.398 (7)	C33—H33	0.9300
C4—H4	0.9300	C34—H34A	0.9600
C4—C5	1.354 (7)	C34—H34B	0.9600
C5—H5	0.9300	C34—H34C	0.9600
C5—C6	1.404 (6)	C35—H35A	0.9600
C6—C7	1.449 (7)	C35—H35B	0.9600
C7—H7	0.9300	C35—H35C	0.9600
			
O1—Ce1—Cu1	92.67 (6)	C4—C3—H3	120.0
O2—Ce1—Cu1	32.50 (6)	C3—C4—H4	120.2
O2—Ce1—O1	60.44 (9)	C5—C4—C3	119.6 (5)
O2—Ce1—O4	120.15 (9)	C5—C4—H4	120.2
O2—Ce1—O5	136.52 (10)	C4—C5—H5	119.3
O2—Ce1—O10	101.94 (9)	C4—C5—C6	121.4 (5)
O2—Ce1—O16	75.19 (10)	C6—C5—H5	119.3
O3—Ce1—Cu1	32.68 (6)	C1—C6—C5	120.1 (5)
O3—Ce1—O1	120.41 (9)	C1—C6—C7	122.3 (4)
O3—Ce1—O2	63.72 (9)	C5—C6—C7	117.6 (4)
O3—Ce1—O4	61.22 (9)	N1—C7—C6	128.2 (4)
O3—Ce1—O5	133.40 (10)	N1—C7—H7	115.9
O3—Ce1—O7	78.73 (10)	C6—C7—H7	115.9
O3—Ce1—O10	145.94 (10)	N1—C8—H8A	109.3
O3—Ce1—O16	72.65 (10)	N1—C8—H8B	109.3
O4—Ce1—Cu1	93.41 (6)	H8A—C8—H8B	108.0
O4—Ce1—O1	139.91 (10)	C9—C8—N1	111.5 (4)
O5—Ce1—Cu1	153.50 (7)	C9—C8—H8A	109.3
O5—Ce1—O1	81.36 (10)	C9—C8—H8B	109.3
O5—Ce1—O4	75.80 (10)	C8—C9—H9A	108.8
O5—Ce1—O16	142.48 (10)	C8—C9—H9B	108.8
O7—Ce1—Cu1	83.65 (7)	C8—C9—C10	113.9 (5)
O7—Ce1—O1	71.39 (10)	H9A—C9—H9B	107.7
O7—Ce1—O2	78.22 (9)	C10—C9—H9A	108.8
O7—Ce1—O4	69.99 (10)	C10—C9—H9B	108.8
O7—Ce1—O5	69.96 (10)	N2—C10—C9	113.2 (4)
O7—Ce1—O10	130.88 (10)	N2—C10—H10A	108.9
O7—Ce1—O16	147.31 (10)	N2—C10—H10B	108.9
O10—Ce1—Cu1	122.46 (7)	C9—C10—H10A	108.9
O10—Ce1—O1	66.98 (9)	C9—C10—H10B	108.9
O10—Ce1—O4	137.21 (9)	H10A—C10—H10B	107.8
O10—Ce1—O5	78.98 (10)	N2—C11—H11	116.3
O10—Ce1—O16	73.80 (11)	N2—C11—C12	127.4 (4)
O12—Ce1—Cu1	123.41 (7)	C12—C11—H11	116.3
O12—Ce1—O1	136.25 (9)	C13—C12—C11	118.5 (4)
O12—Ce1—O2	147.06 (10)	C17—C12—C11	122.1 (4)
O12—Ce1—O3	102.31 (10)	C17—C12—C13	119.1 (4)
O12—Ce1—O4	68.07 (10)	C12—C13—H13	119.3
O12—Ce1—O5	75.41 (10)	C14—C13—C12	121.4 (5)
O12—Ce1—O7	130.58 (10)	C14—C13—H13	119.3
O12—Ce1—O10	72.46 (10)	C13—C14—H14	120.1
O12—Ce1—O16	72.08 (11)	C13—C14—C15	119.9 (5)
O16—Ce1—Cu1	63.73 (7)	C15—C14—H14	120.1
O16—Ce1—O1	110.17 (10)	C14—C15—H15	120.0
O16—Ce1—O4	108.01 (11)	C16—C15—C14	119.9 (5)
O2—Cu1—Ce1	42.43 (8)	C16—C15—H15	120.0
O2—Cu1—O3	82.27 (11)	O4—C16—C17	114.2 (4)
O2—Cu1—O15	95.26 (13)	C15—C16—O4	124.6 (4)
O2—Cu1—N2	170.36 (14)	C15—C16—C17	121.2 (4)
O3—Cu1—Ce1	41.90 (8)	O3—C17—C12	123.6 (4)
O3—Cu1—O15	91.17 (13)	O3—C17—C16	118.3 (4)
O3—Cu1—N2	89.90 (14)	C12—C17—C16	118.1 (4)
O15—Cu1—Ce1	84.05 (9)	O1—C18—H18A	109.5
N1—Cu1—Ce1	133.99 (12)	O1—C18—H18B	109.5
N1—Cu1—O2	91.93 (14)	O1—C18—H18C	109.5
N1—Cu1—O3	169.67 (15)	H18A—C18—H18B	109.5
N1—Cu1—O15	97.89 (15)	H18A—C18—H18C	109.5
N1—Cu1—N2	94.94 (16)	H18B—C18—H18C	109.5
N2—Cu1—Ce1	131.06 (11)	O4—C19—H19A	109.5
N2—Cu1—O15	90.50 (15)	O4—C19—H19B	109.5
O5—S1—N3	113.1 (2)	O4—C19—H19C	109.5
O5—S1—C20	104.0 (2)	H19A—C19—H19B	109.5
O6—S1—O5	112.9 (2)	H19A—C19—H19C	109.5
O6—S1—N3	111.8 (2)	H19B—C19—H19C	109.5
O6—S1—C20	107.0 (2)	C21—C20—S1	116.7 (4)
N3—S1—C20	107.4 (2)	C25—C20—S1	122.5 (4)
O10—S2—N4	113.1 (2)	C25—C20—C21	120.7 (5)
O10—S2—C28	106.0 (2)	C20—C21—H21	120.4
O11—S2—O10	112.6 (2)	C22—C21—C20	119.3 (5)
O11—S2—N4	113.9 (2)	C22—C21—H21	120.4
O11—S2—C28	106.1 (2)	C21—C22—H22	119.8
N4—S2—C28	104.3 (2)	C23—C22—C21	120.3 (6)
O7—P1—O8	111.03 (19)	C23—C22—H22	119.8
O7—P1—O9	113.06 (19)	C22—C23—H23	119.3
O7—P1—N3	118.4 (2)	C22—C23—C24	121.4 (6)
O8—P1—N3	107.8 (2)	C24—C23—H23	119.3
O9—P1—O8	100.2 (2)	C23—C24—H24	120.1
O9—P1—N3	104.7 (2)	C23—C24—C25	119.8 (6)
O12—P2—O13	113.0 (2)	C25—C24—H24	120.1
O12—P2—O14	111.74 (19)	C20—C25—C24	118.5 (6)
O12—P2—N4	115.41 (19)	C20—C25—H25	120.8
O13—P2—O14	97.1 (2)	C24—C25—H25	120.8
O13—P2—N4	107.8 (2)	O8—C26—H26A	109.5
O14—P2—N4	110.3 (2)	O8—C26—H26B	109.5
C2—O1—Ce1	119.0 (2)	O8—C26—H26C	109.5
C2—O1—C18	115.5 (4)	H26A—C26—H26B	109.5
C18—O1—Ce1	124.5 (3)	H26A—C26—H26C	109.5
Cu1—O2—Ce1	105.07 (12)	H26B—C26—H26C	109.5
C1—O2—Ce1	127.8 (2)	O9—C27—H27A	109.5
C1—O2—Cu1	126.4 (3)	O9—C27—H27B	109.5
Cu1—O3—Ce1	105.42 (11)	O9—C27—H27C	109.5
C17—O3—Ce1	126.1 (2)	H27A—C27—H27B	109.5
C17—O3—Cu1	124.4 (2)	H27A—C27—H27C	109.5
C16—O4—Ce1	118.1 (2)	H27B—C27—H27C	109.5
C16—O4—C19	116.3 (3)	C29—C28—S2	121.7 (4)
C19—O4—Ce1	125.6 (3)	C33—C28—S2	117.8 (4)
S1—O5—Ce1	147.19 (19)	C33—C28—C29	120.4 (4)
P1—O7—Ce1	140.08 (18)	C28—C29—H29	120.7
C26—O8—P1	119.7 (3)	C28—C29—C30	118.7 (5)
C27—O9—P1	120.0 (3)	C30—C29—H29	120.7
S2—O10—Ce1	139.09 (18)	C29—C30—H30	119.7
P2—O12—Ce1	141.39 (18)	C31—C30—C29	120.6 (5)
C34—O13—P2	121.5 (3)	C31—C30—H30	119.7
C35—O14—P2	118.2 (3)	C30—C31—H31	120.0
N5—O15—Cu1	125.4 (3)	C30—C31—C32	120.0 (5)
N5—O16—Ce1	145.5 (3)	C32—C31—H31	120.0
C7—N1—Cu1	125.1 (3)	C31—C32—H32	120.1
C7—N1—C8	115.3 (4)	C33—C32—C31	119.9 (5)
C8—N1—Cu1	119.6 (3)	C33—C32—H32	120.1
C10—N2—Cu1	121.8 (3)	C28—C33—H33	119.8
C11—N2—Cu1	123.3 (3)	C32—C33—C28	120.4 (5)
C11—N2—C10	114.9 (4)	C32—C33—H33	119.8
S1—N3—P1	128.5 (3)	O13—C34—H34A	109.5
S2—N4—P2	127.6 (3)	O13—C34—H34B	109.5
O15—N5—O16	119.5 (4)	O13—C34—H34C	109.5
O17—N5—O15	120.2 (4)	H34A—C34—H34B	109.5
O17—N5—O16	120.3 (4)	H34A—C34—H34C	109.5
O2—C1—C2	118.9 (4)	H34B—C34—H34C	109.5
O2—C1—C6	123.9 (4)	O14—C35—H35A	109.5
C6—C1—C2	117.2 (4)	O14—C35—H35B	109.5
O1—C2—C1	113.7 (4)	O14—C35—H35C	109.5
C3—C2—O1	124.6 (4)	H35A—C35—H35B	109.5
C3—C2—C1	121.7 (4)	H35A—C35—H35C	109.5
C2—C3—H3	120.0	H35B—C35—H35C	109.5
C2—C3—C4	120.0 (5)		
			
Ce1—O1—C2—C1	4.8 (4)	N1—C8—C9—C10	−77.3 (6)
Ce1—O1—C2—C3	−177.1 (3)	N2—C11—C12—C13	−172.3 (5)
Ce1—O2—C1—C2	0.0 (5)	N2—C11—C12—C17	14.3 (7)
Ce1—O2—C1—C6	−177.7 (3)	N3—S1—O5—Ce1	−7.2 (4)
Ce1—O3—C17—C12	−173.8 (3)	N3—S1—C20—C21	170.4 (4)
Ce1—O3—C17—C16	9.1 (5)	N3—S1—C20—C25	−5.7 (5)
Ce1—O4—C16—C15	166.3 (4)	N3—P1—O7—Ce1	−24.2 (4)
Ce1—O4—C16—C17	−13.2 (5)	N3—P1—O8—C26	−81.9 (4)
Ce1—O16—N5—O15	−20.1 (8)	N3—P1—O9—C27	−171.4 (4)
Ce1—O16—N5—O17	161.0 (4)	N4—S2—O10—Ce1	35.3 (4)
Cu1—O2—C1—C2	−168.1 (3)	N4—S2—C28—C29	−5.6 (5)
Cu1—O2—C1—C6	14.2 (6)	N4—S2—C28—C33	177.4 (4)
Cu1—O3—C17—C12	−19.8 (5)	N4—P2—O12—Ce1	9.4 (4)
Cu1—O3—C17—C16	163.0 (3)	N4—P2—O13—C34	−47.5 (5)
Cu1—O15—N5—O16	14.6 (6)	N4—P2—O14—C35	66.7 (4)
Cu1—O15—N5—O17	−166.5 (3)	C1—C2—C3—C4	3.2 (7)
Cu1—N1—C7—C6	−0.1 (7)	C1—C6—C7—N1	−7.5 (8)
Cu1—N1—C8—C9	31.3 (6)	C2—C1—C6—C5	1.7 (6)
Cu1—N2—C10—C9	2.5 (6)	C2—C1—C6—C7	−178.0 (4)
Cu1—N2—C11—C12	9.0 (7)	C2—C3—C4—C5	−0.6 (7)
S1—C20—C21—C22	−176.2 (5)	C3—C4—C5—C6	−1.4 (8)
S1—C20—C25—C24	175.7 (5)	C4—C5—C6—C1	0.8 (7)
S2—C28—C29—C30	−177.5 (4)	C4—C5—C6—C7	−179.5 (5)
S2—C28—C33—C32	178.6 (5)	C5—C6—C7—N1	172.8 (5)
O1—C2—C3—C4	−174.8 (4)	C6—C1—C2—O1	174.5 (4)
O2—C1—C2—O1	−3.3 (5)	C6—C1—C2—C3	−3.7 (6)
O2—C1—C2—C3	178.5 (4)	C7—N1—C8—C9	−149.7 (5)
O2—C1—C6—C5	179.4 (4)	C8—N1—C7—C6	−179.0 (5)
O2—C1—C6—C7	−0.3 (7)	C8—C9—C10—N2	57.6 (6)
O4—C16—C17—O3	3.8 (5)	C10—N2—C11—C12	−172.8 (4)
O4—C16—C17—C12	−173.5 (4)	C11—N2—C10—C9	−175.7 (4)
O5—S1—N3—P1	−0.2 (5)	C11—C12—C13—C14	−174.5 (5)
O5—S1—C20—C21	50.2 (4)	C11—C12—C17—O3	−8.2 (6)
O5—S1—C20—C25	−125.9 (5)	C11—C12—C17—C16	169.0 (4)
O6—S1—O5—Ce1	−135.3 (3)	C12—C13—C14—C15	3.7 (8)
O6—S1—N3—P1	128.5 (4)	C13—C12—C17—O3	178.4 (4)
O6—S1—C20—C21	−69.5 (4)	C13—C12—C17—C16	−4.4 (6)
O6—S1—C20—C25	114.4 (5)	C13—C14—C15—C16	−1.1 (8)
O7—P1—O8—C26	49.3 (5)	C14—C15—C16—O4	176.3 (4)
O7—P1—O9—C27	58.4 (4)	C14—C15—C16—C17	−4.3 (7)
O7—P1—N3—S1	12.9 (5)	C15—C16—C17—O3	−175.7 (4)
O8—P1—O7—Ce1	−149.6 (3)	C15—C16—C17—C12	7.0 (6)
O8—P1—O9—C27	−59.8 (4)	C17—C12—C13—C14	−0.9 (7)
O8—P1—N3—S1	139.9 (3)	C18—O1—C2—C1	−164.2 (4)
O9—P1—O7—Ce1	98.7 (3)	C18—O1—C2—C3	13.9 (6)
O9—P1—O8—C26	169.0 (4)	C19—O4—C16—C15	−12.0 (6)
O9—P1—N3—S1	−114.1 (4)	C19—O4—C16—C17	168.6 (4)
O10—S2—N4—P2	−39.9 (4)	C20—S1—O5—Ce1	109.1 (4)
O10—S2—C28—C29	−125.2 (4)	C20—S1—N3—P1	−114.5 (4)
O10—S2—C28—C33	57.7 (4)	C20—C21—C22—C23	0.8 (10)
O11—S2—O10—Ce1	−95.5 (3)	C21—C20—C25—C24	−0.2 (8)
O11—S2—N4—P2	90.3 (4)	C21—C22—C23—C24	−1.5 (11)
O11—S2—C28—C29	114.9 (4)	C22—C23—C24—C25	1.3 (11)
O11—S2—C28—C33	−62.1 (4)	C23—C24—C25—C20	−0.4 (10)
O12—P2—O13—C34	81.2 (4)	C25—C20—C21—C22	0.0 (8)
O12—P2—O14—C35	−63.1 (4)	C28—S2—O10—Ce1	149.0 (3)
O12—P2—N4—S2	21.6 (5)	C28—S2—N4—P2	−154.6 (3)
O13—P2—O12—Ce1	−115.2 (3)	C28—C29—C30—C31	−1.2 (8)
O13—P2—O14—C35	178.7 (4)	C29—C28—C33—C32	1.5 (8)
O13—P2—N4—S2	148.9 (3)	C29—C30—C31—C32	1.9 (8)
O14—P2—O12—Ce1	136.4 (3)	C30—C31—C32—C33	−1.0 (9)
O14—P2—O13—C34	−161.5 (4)	C31—C32—C33—C28	−0.7 (9)
O14—P2—N4—S2	−106.2 (4)	C33—C28—C29—C30	−0.5 (7)

### [µ-Dimethyl (phenylsulfonyl)amidophosphato][dimethyl(phenylsulfonyl)amidophosphato](nitrato)(µ-6,6'-{(1E,1'E)-[propane-1,3-diylbis(azanylylidene)]bis(methanylylidene)}bis(2-methoxyphenolato))gadolinium(III)copper(II) (2) Crystal data

**Table:**

[CuGd(C_19_H_20_N_2_O_4_)(C_8_H_11_NO_5_PS)_2_(NO_3_)]	*F*(000) = 2312
*M**_r_* = 1151.58	*D*_x_ = 1.722 Mg m^−^^3^
Monoclinic, *P*2_1_/*n*	Mo *K*α radiation, λ = 0.71073 Å
*a* = 9.9250 (2) Å	Cell parameters from 14971 reflections
*b* = 16.3468 (4) Å	θ = 3.0–30.9°
*c* = 27.4479 (6) Å	µ = 2.20 mm^−^^1^
β = 93.978 (2)°	*T* = 293 K
*V* = 4442.47 (17) Å^3^	Block, colourless
*Z* = 4	0.3 × 0.2 × 0.1 mm

### [µ-Dimethyl (phenylsulfonyl)amidophosphato][dimethyl(phenylsulfonyl)amidophosphato](nitrato)(µ-6,6'-{(1E,1'E)-[propane-1,3-diylbis(azanylylidene)]bis(methanylylidene)}bis(2-methoxyphenolato))gadolinium(III)copper(II) (2) Data collection

**Table:**

Bruker APEXII CCD diffractometer	7249 reflections with *I* > 2σ(*I*)
Detector resolution: 16.1827 pixels mm^-1^	*R*_int_ = 0.038
φ and ω scans	θ_max_ = 26.0°, θ_min_ = 2.9°
Absorption correction: numerical (CrysAlisPro; Rigaku OD, 2024)	*h* = −11→12
*T*_min_ = 0.111, *T*_max_ = 0.342	*k* = −19→20
38125 measured reflections	*l* = −33→33
8728 independent reflections	

### [µ-Dimethyl (phenylsulfonyl)amidophosphato][dimethyl(phenylsulfonyl)amidophosphato](nitrato)(µ-6,6'-{(1E,1'E)-[propane-1,3-diylbis(azanylylidene)]bis(methanylylidene)}bis(2-methoxyphenolato))gadolinium(III)copper(II) (2) Refinement

**Table:**

Refinement on *F*^2^	60 restraints
Least-squares matrix: full	Hydrogen site location: inferred from neighbouring sites
*R*[*F*^2^ > 2σ(*F*^2^)] = 0.038	H-atom parameters constrained
*wR*(*F*^2^) = 0.091	*w* = 1/[σ^2^(*F*_o_^2^) + (0.0411*P*)^2^ + 4.4598*P*] where *P* = (*F*_o_^2^ + 2*F*_c_^2^)/3
*S* = 1.07	(Δ/σ)_max_ = 0.002
8728 reflections	Δρ_max_ = 0.91 e Å^−^^3^
550 parameters	Δρ_min_ = −0.51 e Å^−^^3^

### [µ-Dimethyl (phenylsulfonyl)amidophosphato][dimethyl(phenylsulfonyl)amidophosphato](nitrato)(µ-6,6'-{(1E,1'E)-[propane-1,3-diylbis(azanylylidene)]bis(methanylylidene)}bis(2-methoxyphenolato))gadolinium(III)copper(II) (2) Special details

**Table:**

Geometry. All esds (except the esd in the dihedral angle between two l.s. planes) are estimated using the full covariance matrix. The cell esds are taken into account individually in the estimation of esds in distances, angles and torsion angles; correlations between esds in cell parameters are only used when they are defined by crystal symmetry. An approximate (isotropic) treatment of cell esds is used for estimating esds involving l.s. planes.

### [µ-Dimethyl (phenylsulfonyl)amidophosphato][dimethyl(phenylsulfonyl)amidophosphato](nitrato)(µ-6,6'-{(1E,1'E)-[propane-1,3-diylbis(azanylylidene)]bis(methanylylidene)}bis(2-methoxyphenolato))gadolinium(III)copper(II) (2) Fractional atomic coordinates and isotropic or equivalent isotropic displacement parameters (Å^2^)

**Table:**

	*x*	*y*	*z*	*U*_iso_*/*U*_eq_	
Gd1	0.64031 (2)	0.67813 (2)	0.37827 (2)	0.03558 (7)	
Cu1	0.47238 (5)	0.75095 (3)	0.47727 (2)	0.03861 (13)	
S1	0.80633 (11)	0.83029 (7)	0.53310 (4)	0.0485 (3)	
S2	0.64980 (12)	0.47850 (7)	0.31157 (5)	0.0509 (3)	
P1	0.91846 (11)	0.79475 (8)	0.44363 (4)	0.0446 (3)	
P2	0.83478 (13)	0.59615 (8)	0.28371 (5)	0.0564 (3)	
O1	0.7717 (3)	0.56841 (19)	0.42903 (11)	0.0513 (8)	
O2	0.5707 (3)	0.65363 (17)	0.45722 (10)	0.0400 (6)	
O3	0.5282 (3)	0.78897 (16)	0.41417 (10)	0.0410 (6)	
O4	0.6673 (3)	0.81962 (17)	0.33865 (11)	0.0515 (8)	
O5	0.6751 (3)	0.7989 (3)	0.51847 (14)	0.0731 (11)	
O6	0.8092 (4)	0.9167 (2)	0.54180 (15)	0.0794 (11)	
O7	0.8252 (3)	0.7374 (2)	0.41797 (12)	0.0611 (9)	
O8	0.8943 (4)	0.8781 (2)	0.41631 (14)	0.0664 (9)	
O9	1.0696 (3)	0.7704 (2)	0.43748 (12)	0.0635 (9)	
O10	0.5960 (3)	0.53868 (18)	0.34445 (11)	0.0484 (7)	
O11	0.7052 (4)	0.4080 (2)	0.33629 (16)	0.0847 (12)	
O12	0.7958 (3)	0.65455 (19)	0.32100 (12)	0.0511 (8)	
O13	0.9883 (4)	0.5747 (3)	0.29987 (19)	0.0975 (15)	
O14	0.8489 (5)	0.6357 (3)	0.23357 (15)	0.0860 (12)	
O15	0.4876 (3)	0.6965 (2)	0.30392 (12)	0.0625 (9)	
O16	0.3917 (3)	0.6501 (2)	0.36634 (12)	0.0511 (8)	
O17	0.2761 (4)	0.6643 (3)	0.29725 (17)	0.0890 (13)	
N1	0.4011 (4)	0.6892 (2)	0.53126 (14)	0.0529 (10)	
N2	0.3708 (3)	0.8532 (2)	0.48847 (13)	0.0423 (8)	
N3	0.9191 (4)	0.8026 (3)	0.50065 (15)	0.0631 (11)	
N4	0.7467 (4)	0.5170 (3)	0.27592 (15)	0.0606 (11)	
N5	0.3819 (4)	0.6703 (2)	0.32192 (16)	0.0534 (10)	
C1	0.6230 (4)	0.5982 (2)	0.48868 (15)	0.0389 (9)	
C2	0.7308 (4)	0.5503 (3)	0.47461 (16)	0.0443 (10)	
C3	0.7866 (5)	0.4905 (3)	0.50448 (19)	0.0585 (13)	
H3	0.858589	0.459710	0.494549	0.070*	
C4	0.7363 (5)	0.4756 (3)	0.5495 (2)	0.0684 (15)	
H4	0.773961	0.434735	0.569671	0.082*	
C5	0.6319 (5)	0.5209 (3)	0.56406 (19)	0.0597 (13)	
H5	0.598213	0.510436	0.594219	0.072*	
C6	0.5737 (4)	0.5834 (3)	0.53441 (16)	0.0459 (10)	
C7	0.4594 (5)	0.6260 (3)	0.55063 (17)	0.0536 (12)	
H7	0.423111	0.605498	0.578483	0.064*	
C8	0.2782 (6)	0.7187 (4)	0.5539 (2)	0.0712 (15)	
H8A	0.200271	0.712571	0.530820	0.085*	
H8B	0.263225	0.685864	0.582445	0.085*	
C9	0.2932 (6)	0.8052 (4)	0.5682 (2)	0.0720 (16)	
H9A	0.382616	0.813211	0.584065	0.086*	
H9B	0.227944	0.817787	0.591806	0.086*	
C10	0.2743 (5)	0.8632 (3)	0.52686 (18)	0.0563 (12)	
H10A	0.282257	0.918452	0.539570	0.068*	
H10B	0.183350	0.856788	0.512013	0.068*	
C11	0.3864 (4)	0.9182 (3)	0.46350 (17)	0.0467 (10)	
H11	0.341528	0.964403	0.473737	0.056*	
C12	0.4645 (4)	0.9292 (3)	0.42150 (16)	0.0450 (10)	
C13	0.4677 (6)	1.0090 (3)	0.4022 (2)	0.0643 (14)	
H13	0.422754	1.051151	0.417048	0.077*	
C14	0.5357 (6)	1.0249 (3)	0.3621 (2)	0.0713 (16)	
H14	0.535853	1.077605	0.349353	0.086*	
C15	0.6054 (5)	0.9627 (3)	0.33985 (18)	0.0584 (13)	
H15	0.654161	0.974218	0.312930	0.070*	
C16	0.6019 (4)	0.8849 (3)	0.35772 (15)	0.0423 (9)	
C17	0.5298 (4)	0.8659 (2)	0.39907 (15)	0.0384 (9)	
C18	0.9005 (6)	0.5389 (5)	0.4174 (2)	0.100 (3)	
H18A	0.924541	0.563174	0.387371	0.150*	
H18B	0.897104	0.480528	0.413912	0.150*	
H18C	0.966781	0.553222	0.443155	0.150*	
C19	0.7594 (7)	0.8389 (3)	0.3025 (2)	0.084 (2)	
H19A	0.811776	0.791308	0.295897	0.126*	
H19B	0.818501	0.882100	0.314298	0.126*	
H19C	0.709653	0.856210	0.273062	0.126*	
C20	0.8533 (4)	0.7841 (2)	0.58896 (12)	0.0548 (12)	
C25	0.8349 (5)	0.8272 (2)	0.63167 (16)	0.108 (2)	
H25	0.799757	0.879917	0.630071	0.129*	
C24	0.8690 (7)	0.7913 (4)	0.67678 (12)	0.130 (3)	
H24	0.856675	0.820134	0.705356	0.156*	
C23	0.9215 (6)	0.7125 (4)	0.67917 (15)	0.137 (3)	
H23	0.944287	0.688539	0.709353	0.164*	
C22	0.9399 (7)	0.6695 (3)	0.6365 (2)	0.148 (4)	
H22	0.974980	0.616727	0.638064	0.178*	
C21	0.9058 (6)	0.7053 (2)	0.59135 (16)	0.109 (2)	
H21	0.918062	0.676508	0.562778	0.131*	
C26	0.9671 (7)	0.9501 (4)	0.4304 (2)	0.0883 (19)	
H26A	0.926714	0.996343	0.413449	0.132*	
H26B	1.059141	0.944587	0.422253	0.132*	
H26C	0.964511	0.958072	0.464961	0.132*	
C27	1.1119 (6)	0.7546 (4)	0.3901 (2)	0.0813 (18)	
H27A	1.092462	0.801292	0.369582	0.122*	
H27B	1.064624	0.707767	0.376550	0.122*	
H27C	1.207311	0.744127	0.392126	0.122*	
C28	0.5135 (4)	0.4446 (3)	0.27334 (15)	0.0660 (15)	
C29	0.4498 (5)	0.4967 (2)	0.23897 (18)	0.097 (2)	
H29	0.480113	0.550226	0.236045	0.117*	
C30	0.3408 (5)	0.4689 (4)	0.20899 (17)	0.120 (3)	
H30	0.298128	0.503756	0.185995	0.145*	
C31	0.2954 (4)	0.3890 (4)	0.2134 (2)	0.123 (3)	
H31	0.222468	0.370327	0.193303	0.147*	
C32	0.3591 (6)	0.3369 (3)	0.2477 (2)	0.154 (4)	
H32	0.328793	0.283367	0.250661	0.185*	
C33	0.4682 (5)	0.3647 (2)	0.27772 (18)	0.122 (3)	
H33	0.510778	0.329836	0.300711	0.147*	
C34	1.0657 (7)	0.5189 (5)	0.2799 (3)	0.110 (3)	
H34A	1.101059	0.541227	0.251042	0.164*	
H34B	1.012783	0.471210	0.271394	0.164*	
H34C	1.139070	0.504193	0.302863	0.164*	
C35	0.7307 (10)	0.6673 (6)	0.2082 (3)	0.133 (3)	
H35A	0.694734	0.627366	0.185125	0.200*	
H35B	0.752735	0.716268	0.191164	0.200*	
H35C	0.664589	0.679520	0.231049	0.200*	

### [µ-Dimethyl (phenylsulfonyl)amidophosphato][dimethyl(phenylsulfonyl)amidophosphato](nitrato)(µ-6,6'-{(1E,1'E)-[propane-1,3-diylbis(azanylylidene)]bis(methanylylidene)}bis(2-methoxyphenolato))gadolinium(III)copper(II) (2) Atomic displacement parameters (Å^2^)

**Table:**

	*U*^11^	*U*^22^	*U*^33^	*U*^12^	*U*^13^	*U*^23^
Gd1	0.04010 (11)	0.03890 (12)	0.02866 (11)	−0.00117 (9)	0.00897 (8)	−0.00255 (8)
Cu1	0.0464 (3)	0.0393 (3)	0.0316 (3)	−0.0012 (2)	0.0133 (2)	0.0013 (2)
S1	0.0458 (6)	0.0589 (7)	0.0415 (6)	0.0014 (5)	0.0075 (5)	−0.0103 (5)
S2	0.0592 (7)	0.0426 (6)	0.0528 (7)	0.0020 (5)	0.0182 (5)	−0.0074 (5)
P1	0.0388 (6)	0.0555 (7)	0.0403 (7)	−0.0050 (5)	0.0082 (5)	−0.0086 (5)
P2	0.0601 (7)	0.0580 (8)	0.0550 (8)	−0.0027 (6)	0.0317 (6)	−0.0102 (6)
O1	0.0502 (17)	0.062 (2)	0.0423 (18)	0.0152 (15)	0.0066 (14)	0.0030 (15)
O2	0.0490 (16)	0.0400 (15)	0.0319 (16)	0.0021 (13)	0.0085 (12)	0.0038 (12)
O3	0.0571 (17)	0.0347 (14)	0.0332 (16)	−0.0010 (13)	0.0161 (13)	0.0040 (12)
O4	0.074 (2)	0.0420 (16)	0.0409 (18)	−0.0068 (15)	0.0243 (15)	0.0035 (13)
O5	0.0442 (18)	0.115 (3)	0.060 (2)	−0.0084 (19)	0.0017 (16)	−0.019 (2)
O6	0.103 (3)	0.058 (2)	0.079 (3)	0.009 (2)	0.019 (2)	−0.004 (2)
O7	0.0553 (19)	0.074 (2)	0.054 (2)	−0.0148 (17)	0.0087 (15)	−0.0167 (17)
O8	0.073 (2)	0.058 (2)	0.067 (2)	−0.0088 (18)	−0.0037 (18)	−0.0027 (18)
O9	0.0472 (18)	0.095 (3)	0.050 (2)	0.0061 (18)	0.0125 (15)	−0.0102 (19)
O10	0.0550 (17)	0.0490 (17)	0.0432 (18)	−0.0049 (14)	0.0176 (14)	−0.0093 (14)
O11	0.104 (3)	0.058 (2)	0.094 (3)	0.028 (2)	0.017 (2)	0.012 (2)
O12	0.0530 (17)	0.0539 (18)	0.0492 (19)	−0.0065 (14)	0.0235 (14)	−0.0088 (15)
O13	0.056 (2)	0.105 (3)	0.135 (4)	0.004 (2)	0.028 (2)	−0.024 (3)
O14	0.110 (3)	0.097 (3)	0.055 (3)	−0.006 (3)	0.038 (2)	−0.010 (2)
O15	0.065 (2)	0.078 (2)	0.043 (2)	−0.0132 (18)	0.0002 (16)	0.0159 (17)
O16	0.0454 (17)	0.0623 (19)	0.046 (2)	0.0065 (15)	0.0107 (14)	−0.0005 (15)
O17	0.062 (2)	0.117 (4)	0.084 (3)	−0.001 (2)	−0.025 (2)	0.011 (3)
N1	0.064 (2)	0.052 (2)	0.045 (2)	−0.0016 (19)	0.0235 (19)	0.0057 (18)
N2	0.0422 (19)	0.048 (2)	0.037 (2)	0.0015 (16)	0.0054 (15)	−0.0045 (16)
N3	0.050 (2)	0.098 (3)	0.042 (2)	0.007 (2)	0.0066 (18)	−0.009 (2)
N4	0.070 (3)	0.065 (3)	0.051 (3)	−0.007 (2)	0.031 (2)	−0.022 (2)
N5	0.052 (2)	0.055 (2)	0.052 (3)	0.0042 (19)	−0.004 (2)	−0.0018 (19)
C1	0.041 (2)	0.040 (2)	0.035 (2)	−0.0057 (18)	−0.0011 (17)	0.0020 (18)
C2	0.044 (2)	0.050 (3)	0.038 (2)	−0.006 (2)	−0.0029 (18)	0.0053 (19)
C3	0.051 (3)	0.061 (3)	0.062 (3)	0.012 (2)	−0.003 (2)	0.012 (3)
C4	0.066 (3)	0.074 (4)	0.064 (4)	0.004 (3)	−0.003 (3)	0.033 (3)
C5	0.059 (3)	0.070 (3)	0.050 (3)	−0.009 (3)	0.006 (2)	0.020 (3)
C6	0.054 (3)	0.047 (2)	0.037 (2)	−0.009 (2)	0.0039 (19)	0.0097 (19)
C7	0.071 (3)	0.055 (3)	0.037 (3)	−0.010 (2)	0.018 (2)	0.009 (2)
C8	0.078 (4)	0.078 (4)	0.063 (4)	−0.002 (3)	0.041 (3)	0.007 (3)
C9	0.081 (4)	0.085 (4)	0.054 (3)	0.015 (3)	0.035 (3)	0.003 (3)
C10	0.054 (3)	0.061 (3)	0.056 (3)	0.009 (2)	0.021 (2)	−0.001 (2)
C11	0.049 (2)	0.042 (2)	0.049 (3)	0.011 (2)	0.007 (2)	−0.005 (2)
C12	0.055 (3)	0.039 (2)	0.042 (3)	0.001 (2)	0.011 (2)	0.0026 (19)
C13	0.088 (4)	0.044 (3)	0.063 (4)	0.014 (3)	0.020 (3)	0.004 (2)
C14	0.104 (4)	0.043 (3)	0.069 (4)	0.010 (3)	0.024 (3)	0.018 (3)
C15	0.080 (3)	0.048 (3)	0.049 (3)	−0.006 (3)	0.021 (2)	0.008 (2)
C16	0.056 (2)	0.041 (2)	0.031 (2)	−0.002 (2)	0.0063 (18)	−0.0002 (18)
C17	0.044 (2)	0.038 (2)	0.033 (2)	−0.0022 (18)	0.0042 (17)	0.0022 (17)
C18	0.076 (4)	0.163 (7)	0.065 (4)	0.063 (4)	0.028 (3)	0.037 (4)
C19	0.132 (5)	0.058 (3)	0.070 (4)	−0.001 (3)	0.064 (4)	0.008 (3)
C20	0.057 (3)	0.059 (3)	0.049 (3)	−0.002 (2)	0.008 (2)	−0.009 (2)
C25	0.162 (6)	0.099 (5)	0.060 (4)	0.020 (4)	−0.005 (4)	−0.012 (3)
C24	0.189 (7)	0.132 (6)	0.069 (5)	0.009 (6)	0.001 (5)	−0.009 (4)
C23	0.166 (7)	0.146 (7)	0.096 (6)	0.020 (6)	−0.005 (5)	0.028 (5)
C22	0.188 (7)	0.141 (6)	0.120 (6)	0.072 (6)	0.042 (6)	0.043 (5)
C21	0.149 (6)	0.107 (5)	0.077 (5)	0.050 (5)	0.036 (4)	0.014 (4)
C26	0.113 (5)	0.062 (4)	0.089 (5)	−0.020 (4)	0.008 (4)	−0.004 (3)
C27	0.065 (3)	0.121 (5)	0.061 (4)	0.010 (3)	0.027 (3)	−0.015 (3)
C28	0.071 (3)	0.064 (3)	0.066 (4)	−0.014 (3)	0.030 (3)	−0.030 (3)
C29	0.073 (4)	0.106 (5)	0.109 (5)	0.002 (4)	−0.017 (4)	−0.023 (4)
C30	0.093 (5)	0.145 (6)	0.122 (6)	0.017 (5)	0.000 (4)	−0.031 (5)
C31	0.097 (5)	0.152 (7)	0.121 (6)	−0.027 (5)	0.017 (4)	−0.062 (5)
C32	0.163 (7)	0.133 (6)	0.165 (8)	−0.072 (6)	0.006 (6)	−0.042 (6)
C33	0.143 (6)	0.103 (5)	0.120 (6)	−0.060 (5)	0.011 (5)	−0.025 (4)
C34	0.075 (4)	0.116 (6)	0.137 (7)	0.020 (4)	0.005 (4)	−0.025 (5)
C35	0.155 (9)	0.165 (9)	0.080 (6)	0.012 (7)	0.014 (6)	0.004 (5)

### [µ-Dimethyl (phenylsulfonyl)amidophosphato][dimethyl(phenylsulfonyl)amidophosphato](nitrato)(µ-6,6'-{(1E,1'E)-[propane-1,3-diylbis(azanylylidene)]bis(methanylylidene)}bis(2-methoxyphenolato))gadolinium(III)copper(II) (2) Geometric parameters (Å, º)

**Table:**

Gd1—Cu1	3.4939 (5)	C8—H8A	0.9700
Gd1—O1	2.570 (3)	C8—H8B	0.9700
Gd1—O2	2.354 (3)	C8—C9	1.473 (8)
Gd1—O3	2.376 (3)	C9—H9A	0.9700
Gd1—O4	2.578 (3)	C9—H9B	0.9700
Gd1—O7	2.284 (3)	C9—C10	1.480 (7)
Gd1—O10	2.489 (3)	C10—H10A	0.9700
Gd1—O12	2.311 (3)	C10—H10B	0.9700
Gd1—O15	2.475 (3)	C11—H11	0.9300
Gd1—O16	2.508 (3)	C11—C12	1.445 (6)
Gd1—N5	2.904 (4)	C12—C13	1.410 (6)
Cu1—O2	1.965 (3)	C12—C17	1.388 (6)
Cu1—O3	1.956 (3)	C13—H13	0.9300
Cu1—O5	2.372 (3)	C13—C14	1.355 (7)
Cu1—N1	1.965 (4)	C14—H14	0.9300
Cu1—N2	1.986 (4)	C14—C15	1.394 (7)
S1—O5	1.431 (3)	C15—H15	0.9300
S1—O6	1.433 (4)	C15—C16	1.364 (6)
S1—N3	1.546 (4)	C16—C17	1.418 (6)
S1—C20	1.743 (3)	C18—H18A	0.9600
S2—O10	1.461 (3)	C18—H18B	0.9600
S2—O11	1.428 (4)	C18—H18C	0.9600
S2—N4	1.552 (4)	C19—H19A	0.9600
S2—C28	1.744 (3)	C19—H19B	0.9600
P1—O7	1.463 (3)	C19—H19C	0.9600
P1—O8	1.566 (4)	C20—C25	1.3900
P1—O9	1.573 (3)	C20—C21	1.3900
P1—N3	1.570 (4)	C25—H25	0.9300
P2—O12	1.471 (3)	C25—C24	1.3900
P2—O13	1.596 (4)	C24—H24	0.9300
P2—O14	1.536 (4)	C24—C23	1.3900
P2—N4	1.568 (4)	C23—H23	0.9300
O1—C2	1.374 (5)	C23—C22	1.3900
O1—C18	1.423 (6)	C22—H22	0.9300
O2—C1	1.332 (5)	C22—C21	1.3900
O3—C17	1.324 (5)	C21—H21	0.9300
O4—C16	1.372 (5)	C26—H26A	0.9600
O4—C19	1.430 (6)	C26—H26B	0.9600
O8—C26	1.420 (6)	C26—H26C	0.9600
O9—C27	1.417 (6)	C27—H27A	0.9600
O13—C34	1.334 (7)	C27—H27B	0.9600
O14—C35	1.419 (9)	C27—H27C	0.9600
O15—N5	1.265 (5)	C28—C29	1.3900
O16—N5	1.260 (5)	C28—C33	1.3900
O17—N5	1.214 (5)	C29—H29	0.9300
N1—C7	1.281 (6)	C29—C30	1.3900
N1—C8	1.487 (6)	C30—H30	0.9300
N2—C10	1.481 (5)	C30—C31	1.3900
N2—C11	1.280 (6)	C31—H31	0.9300
C1—C2	1.401 (6)	C31—C32	1.3900
C1—C6	1.399 (6)	C32—H32	0.9300
C2—C3	1.368 (6)	C32—C33	1.3900
C3—H3	0.9300	C33—H33	0.9300
C3—C4	1.386 (7)	C34—H34A	0.9600
C4—H4	0.9300	C34—H34B	0.9600
C4—C5	1.355 (7)	C34—H34C	0.9600
C5—H5	0.9300	C35—H35A	0.9600
C5—C6	1.405 (6)	C35—H35B	0.9600
C6—C7	1.429 (7)	C35—H35C	0.9600
C7—H7	0.9300		
			
O1—Gd1—Cu1	93.88 (7)	O2—C1—C6	123.7 (4)
O1—Gd1—O4	142.29 (11)	C6—C1—C2	118.1 (4)
O1—Gd1—N5	130.83 (11)	O1—C2—C1	114.2 (4)
O2—Gd1—Cu1	32.41 (7)	C3—C2—O1	124.5 (4)
O2—Gd1—O1	63.41 (9)	C3—C2—C1	121.3 (4)
O2—Gd1—O3	64.67 (9)	C2—C3—H3	119.9
O2—Gd1—O4	125.93 (9)	C2—C3—C4	120.2 (5)
O2—Gd1—O10	97.58 (10)	C4—C3—H3	119.9
O2—Gd1—O15	125.30 (11)	C3—C4—H4	120.1
O2—Gd1—O16	74.95 (10)	C5—C4—C3	119.8 (5)
O2—Gd1—N5	100.27 (11)	C5—C4—H4	120.1
O3—Gd1—Cu1	32.34 (6)	C4—C5—H5	119.4
O3—Gd1—O1	122.82 (10)	C4—C5—C6	121.2 (5)
O3—Gd1—O4	63.65 (9)	C6—C5—H5	119.4
O3—Gd1—O10	141.02 (9)	C1—C6—C5	119.4 (4)
O3—Gd1—O15	88.37 (11)	C1—C6—C7	122.0 (4)
O3—Gd1—O16	73.05 (10)	C5—C6—C7	118.5 (4)
O3—Gd1—N5	80.39 (11)	N1—C7—C6	127.6 (4)
O4—Gd1—Cu1	95.26 (7)	N1—C7—H7	116.2
O4—Gd1—N5	85.92 (11)	C6—C7—H7	116.2
O7—Gd1—Cu1	83.98 (8)	N1—C8—H8A	109.5
O7—Gd1—O1	71.16 (12)	N1—C8—H8B	109.5
O7—Gd1—O2	84.85 (11)	H8A—C8—H8B	108.1
O7—Gd1—O3	82.08 (11)	C9—C8—N1	110.6 (4)
O7—Gd1—O4	73.55 (12)	C9—C8—H8A	109.5
O7—Gd1—O10	133.01 (11)	C9—C8—H8B	109.5
O7—Gd1—O12	80.96 (11)	C8—C9—H9A	108.8
O7—Gd1—O15	140.15 (12)	C8—C9—H9B	108.8
O7—Gd1—O16	152.88 (11)	C8—C9—C10	113.9 (5)
O7—Gd1—N5	157.42 (12)	H9A—C9—H9B	107.7
O10—Gd1—Cu1	121.37 (6)	C10—C9—H9A	108.8
O10—Gd1—O1	68.47 (10)	C10—C9—H9B	108.8
O10—Gd1—O4	133.26 (10)	N2—C10—H10A	108.5
O10—Gd1—O16	68.74 (10)	N2—C10—H10B	108.5
O10—Gd1—N5	68.54 (10)	C9—C10—N2	114.9 (4)
O12—Gd1—Cu1	164.40 (8)	C9—C10—H10A	108.5
O12—Gd1—O1	85.10 (11)	C9—C10—H10B	108.5
O12—Gd1—O2	148.25 (11)	H10A—C10—H10B	107.5
O12—Gd1—O3	139.79 (10)	N2—C11—H11	115.8
O12—Gd1—O4	76.63 (10)	N2—C11—C12	128.5 (4)
O12—Gd1—O10	72.70 (10)	C12—C11—H11	115.8
O12—Gd1—O15	81.90 (12)	C13—C12—C11	116.3 (4)
O12—Gd1—O16	125.33 (11)	C17—C12—C11	123.6 (4)
O12—Gd1—N5	103.72 (12)	C17—C12—C13	120.0 (4)
O15—Gd1—Cu1	107.58 (9)	C12—C13—H13	119.7
O15—Gd1—O1	142.24 (11)	C14—C13—C12	120.5 (5)
O15—Gd1—O4	67.58 (11)	C14—C13—H13	119.7
O15—Gd1—O10	73.83 (11)	C13—C14—H14	119.8
O15—Gd1—O16	51.24 (11)	C13—C14—C15	120.4 (5)
O15—Gd1—N5	25.64 (11)	C15—C14—H14	119.8
O16—Gd1—Cu1	69.25 (7)	C14—C15—H15	120.1
O16—Gd1—O1	113.63 (10)	C16—C15—C14	119.7 (4)
O16—Gd1—O4	103.82 (11)	C16—C15—H15	120.1
O16—Gd1—N5	25.61 (11)	O4—C16—C17	114.4 (3)
N5—Gd1—Cu1	88.74 (9)	C15—C16—O4	124.3 (4)
O2—Cu1—Gd1	39.94 (8)	C15—C16—C17	121.3 (4)
O2—Cu1—O5	88.84 (13)	O3—C17—C12	123.6 (4)
O2—Cu1—N2	172.60 (13)	O3—C17—C16	118.5 (4)
O3—Cu1—Gd1	40.53 (8)	C12—C17—C16	117.9 (4)
O3—Cu1—O2	80.36 (11)	O1—C18—H18A	109.5
O3—Cu1—O5	91.95 (13)	O1—C18—H18B	109.5
O3—Cu1—N1	165.91 (15)	O1—C18—H18C	109.5
O3—Cu1—N2	92.73 (13)	H18A—C18—H18B	109.5
O5—Cu1—Gd1	92.80 (9)	H18A—C18—H18C	109.5
N1—Cu1—Gd1	128.88 (11)	H18B—C18—H18C	109.5
N1—Cu1—O2	90.41 (14)	O4—C19—H19A	109.5
N1—Cu1—O5	98.53 (16)	O4—C19—H19B	109.5
N1—Cu1—N2	95.88 (15)	O4—C19—H19C	109.5
N2—Cu1—Gd1	132.96 (10)	H19A—C19—H19B	109.5
N2—Cu1—O5	94.06 (14)	H19A—C19—H19C	109.5
O5—S1—O6	114.0 (2)	H19B—C19—H19C	109.5
O5—S1—N3	114.5 (2)	C25—C20—S1	118.7 (3)
O5—S1—C20	105.9 (2)	C25—C20—C21	120.0
O6—S1—N3	112.2 (3)	C21—C20—S1	121.3 (3)
O6—S1—C20	106.2 (2)	C20—C25—H25	120.0
N3—S1—C20	102.7 (2)	C24—C25—C20	120.0
O10—S2—N4	112.7 (2)	C24—C25—H25	120.0
O10—S2—C28	106.48 (19)	C25—C24—H24	120.0
O11—S2—O10	113.3 (2)	C25—C24—C23	120.0
O11—S2—N4	113.1 (3)	C23—C24—H24	120.0
O11—S2—C28	106.6 (2)	C24—C23—H23	120.0
N4—S2—C28	103.8 (2)	C24—C23—C22	120.0
O7—P1—O8	104.8 (2)	C22—C23—H23	120.0
O7—P1—O9	111.3 (2)	C23—C22—H22	120.0
O7—P1—N3	119.4 (2)	C21—C22—C23	120.0
O8—P1—O9	106.5 (2)	C21—C22—H22	120.0
O8—P1—N3	113.3 (2)	C20—C21—H21	120.0
N3—P1—O9	100.9 (2)	C22—C21—C20	120.0
O12—P2—O13	104.0 (2)	C22—C21—H21	120.0
O12—P2—O14	113.3 (2)	O8—C26—H26A	109.5
O12—P2—N4	117.38 (19)	O8—C26—H26B	109.5
O14—P2—O13	101.4 (3)	O8—C26—H26C	109.5
O14—P2—N4	108.1 (2)	H26A—C26—H26B	109.5
N4—P2—O13	111.6 (2)	H26A—C26—H26C	109.5
C2—O1—Gd1	118.2 (2)	H26B—C26—H26C	109.5
C2—O1—C18	117.2 (4)	O9—C27—H27A	109.5
C18—O1—Gd1	123.0 (3)	O9—C27—H27B	109.5
Cu1—O2—Gd1	107.65 (12)	O9—C27—H27C	109.5
C1—O2—Gd1	125.9 (2)	H27A—C27—H27B	109.5
C1—O2—Cu1	123.2 (3)	H27A—C27—H27C	109.5
Cu1—O3—Gd1	107.13 (12)	H27B—C27—H27C	109.5
C17—O3—Gd1	125.3 (2)	C29—C28—S2	120.7 (3)
C17—O3—Cu1	126.1 (2)	C29—C28—C33	120.0
C16—O4—Gd1	118.1 (2)	C33—C28—S2	119.3 (3)
C16—O4—C19	115.8 (3)	C28—C29—H29	120.0
C19—O4—Gd1	125.5 (3)	C30—C29—C28	120.0
S1—O5—Cu1	167.9 (2)	C30—C29—H29	120.0
P1—O7—Gd1	164.2 (2)	C29—C30—H30	120.0
C26—O8—P1	122.2 (4)	C29—C30—C31	120.0
C27—O9—P1	119.3 (3)	C31—C30—H30	120.0
S2—O10—Gd1	141.65 (17)	C30—C31—H31	120.0
P2—O12—Gd1	143.31 (19)	C32—C31—C30	120.0
C34—O13—P2	127.0 (5)	C32—C31—H31	120.0
C35—O14—P2	118.0 (5)	C31—C32—H32	120.0
N5—O15—Gd1	96.5 (3)	C31—C32—C33	120.0
N5—O16—Gd1	95.0 (2)	C33—C32—H32	120.0
C7—N1—Cu1	123.4 (3)	C28—C33—H33	120.0
C7—N1—C8	116.6 (4)	C32—C33—C28	120.0
C8—N1—Cu1	119.8 (3)	C32—C33—H33	120.0
C10—N2—Cu1	124.3 (3)	O13—C34—H34A	109.5
C11—N2—Cu1	122.3 (3)	O13—C34—H34B	109.5
C11—N2—C10	113.4 (4)	O13—C34—H34C	109.5
S1—N3—P1	130.2 (3)	H34A—C34—H34B	109.5
S2—N4—P2	127.8 (3)	H34A—C34—H34C	109.5
O15—N5—Gd1	57.9 (2)	H34B—C34—H34C	109.5
O16—N5—Gd1	59.4 (2)	O14—C35—H35A	109.5
O16—N5—O15	117.2 (4)	O14—C35—H35B	109.5
O17—N5—Gd1	177.3 (4)	O14—C35—H35C	109.5
O17—N5—O15	121.2 (4)	H35A—C35—H35B	109.5
O17—N5—O16	121.6 (4)	H35A—C35—H35C	109.5
O2—C1—C2	118.1 (4)	H35B—C35—H35C	109.5
			
Gd1—O1—C2—C1	−2.7 (5)	N1—C8—C9—C10	77.1 (6)
Gd1—O1—C2—C3	176.4 (4)	N2—C11—C12—C13	−177.7 (5)
Gd1—O2—C1—C2	1.2 (5)	N2—C11—C12—C17	4.9 (8)
Gd1—O2—C1—C6	−176.9 (3)	N3—S1—O5—Cu1	−37.0 (14)
Gd1—O3—C17—C12	−179.0 (3)	N3—S1—C20—C25	141.7 (3)
Gd1—O3—C17—C16	1.1 (5)	N3—S1—C20—C21	−39.6 (4)
Gd1—O4—C16—C15	−178.8 (4)	N3—P1—O7—Gd1	86.6 (9)
Gd1—O4—C16—C17	2.2 (5)	N3—P1—O8—C26	48.3 (5)
Gd1—O15—N5—O16	2.6 (4)	N3—P1—O9—C27	178.1 (4)
Gd1—O15—N5—O17	−177.0 (4)	N4—S2—O10—Gd1	−19.4 (4)
Gd1—O16—N5—O15	−2.5 (4)	N4—S2—C28—C29	−51.0 (3)
Gd1—O16—N5—O17	177.0 (4)	N4—S2—C28—C33	129.4 (3)
Cu1—O2—C1—C2	−156.1 (3)	N4—P2—O12—Gd1	−0.2 (5)
Cu1—O2—C1—C6	25.8 (5)	N4—P2—O13—C34	46.6 (7)
Cu1—O3—C17—C12	−14.6 (6)	N4—P2—O14—C35	65.6 (6)
Cu1—O3—C17—C16	165.4 (3)	C1—C2—C3—C4	0.5 (7)
Cu1—N1—C7—C6	−7.6 (7)	C1—C6—C7—N1	−11.7 (8)
Cu1—N1—C8—C9	−51.8 (6)	C2—C1—C6—C5	−1.0 (6)
Cu1—N2—C10—C9	21.9 (6)	C2—C1—C6—C7	−176.4 (4)
Cu1—N2—C11—C12	6.5 (7)	C2—C3—C4—C5	−0.4 (8)
S1—C20—C25—C24	178.8 (3)	C3—C4—C5—C6	−0.4 (8)
S1—C20—C21—C22	−178.8 (4)	C4—C5—C6—C1	1.1 (7)
S2—C28—C29—C30	−179.6 (3)	C4—C5—C6—C7	176.7 (5)
S2—C28—C33—C32	179.6 (3)	C5—C6—C7—N1	172.8 (5)
O1—C2—C3—C4	−178.6 (4)	C6—C1—C2—O1	179.4 (4)
O2—C1—C2—O1	1.2 (5)	C6—C1—C2—C3	0.2 (6)
O2—C1—C2—C3	−178.0 (4)	C7—N1—C8—C9	124.3 (5)
O2—C1—C6—C5	177.1 (4)	C8—N1—C7—C6	176.4 (5)
O2—C1—C6—C7	1.6 (7)	C8—C9—C10—N2	−60.6 (6)
O4—C16—C17—O3	−2.2 (6)	C10—N2—C11—C12	−175.4 (4)
O4—C16—C17—C12	177.9 (4)	C11—N2—C10—C9	−156.1 (5)
O5—S1—N3—P1	41.9 (5)	C11—C12—C13—C14	−178.4 (5)
O5—S1—C20—C25	−97.9 (3)	C11—C12—C17—O3	−0.6 (7)
O5—S1—C20—C21	80.9 (3)	C11—C12—C17—C16	179.3 (4)
O6—S1—O5—Cu1	94.1 (13)	C12—C13—C14—C15	−1.1 (9)
O6—S1—N3—P1	−90.1 (4)	C13—C12—C17—O3	−178.0 (4)
O6—S1—C20—C25	23.7 (4)	C13—C12—C17—C16	1.9 (7)
O6—S1—C20—C21	−157.5 (3)	C13—C14—C15—C16	1.9 (9)
O7—P1—O8—C26	−179.8 (4)	C14—C15—C16—O4	−179.7 (5)
O7—P1—O9—C27	50.3 (5)	C14—C15—C16—C17	−0.8 (8)
O7—P1—N3—S1	−62.1 (5)	C15—C16—C17—O3	178.8 (4)
O8—P1—O7—Gd1	−41.7 (9)	C15—C16—C17—C12	−1.1 (7)
O8—P1—O9—C27	−63.4 (5)	C17—C12—C13—C14	−0.9 (8)
O8—P1—N3—S1	62.1 (5)	C18—O1—C2—C1	163.7 (5)
O9—P1—O7—Gd1	−156.5 (8)	C18—O1—C2—C3	−17.2 (7)
O9—P1—O8—C26	−61.7 (5)	C19—O4—C16—C15	9.4 (7)
O9—P1—N3—S1	175.6 (4)	C19—O4—C16—C17	−169.6 (5)
O10—S2—N4—P2	26.6 (5)	C20—S1—O5—Cu1	−149.5 (13)
O10—S2—C28—C29	68.1 (3)	C20—S1—N3—P1	156.2 (4)
O10—S2—C28—C33	−111.5 (3)	C20—C25—C24—C23	0.0
O11—S2—O10—Gd1	110.5 (3)	C25—C20—C21—C22	0.0
O11—S2—N4—P2	−103.4 (4)	C25—C24—C23—C22	0.0
O11—S2—C28—C29	−170.6 (3)	C24—C23—C22—C21	0.0
O11—S2—C28—C33	9.8 (3)	C23—C22—C21—C20	0.0
O12—P2—O13—C34	174.1 (6)	C21—C20—C25—C24	0.0
O12—P2—O14—C35	−66.2 (6)	C28—S2—O10—Gd1	−132.6 (3)
O12—P2—N4—S2	−19.6 (5)	C28—S2—N4—P2	141.4 (4)
O13—P2—O12—Gd1	−124.0 (4)	C28—C29—C30—C31	0.0
O13—P2—O14—C35	−176.9 (6)	C29—C28—C33—C32	0.0
O13—P2—N4—S2	100.3 (4)	C29—C30—C31—C32	0.0
O14—P2—O12—Gd1	126.9 (4)	C30—C31—C32—C33	0.0
O14—P2—O13—C34	−68.2 (7)	C31—C32—C33—C28	0.0
O14—P2—N4—S2	−149.1 (4)	C33—C28—C29—C30	0.0
